# Mechanism and Intervention of the NPY1R/CREB Signaling Axis in Regulating Inflammatory Response in Aged Ovarian Granulosa Cells and Ovarian Senescence

**DOI:** 10.1096/fj.202601614R

**Published:** 2026-06-09

**Authors:** Junnan Fang, Chaoying Wang, Shuaishuai Guo, Yiping Zhang, Yang Zhang, Huihui Wang, Yue Kong, Haixia Jin, Lin Qi, Yan Liu, Zhaoting Wu, Fuli Zhang, Shuang Wen, Xiaopeng Wang, Guang Yang, Guidong Yao

**Affiliations:** ^1^ Center for Reproductive Medicine The First Affiliated Hospital of Zhengzhou University Zhengzhou China; ^2^ Henan Key Laboratory of Reproduction and Genetics The First Affiliated Hospital of Zhengzhou University Zhengzhou China; ^3^ Clinical Medicine Undergraduate, School of Medicine Zhengzhou University Zhengzhou China; ^4^ NHC Key Laboratory of Birth Defects Prevention Zhengzhou China

**Keywords:** CREB, inflammatory response, NPY1R, oocyte quality, ovarian granulosa cells, ovarian senescence

## Abstract

Ovarian senescence is accompanied by inflammatory responses, yet the underlying mechanism remains incompletely elucidated. In this study, transcriptome sequencing was performed on ovarian granulosa cells from advanced‐age patients and normal (Ctrl) patients, revealing that Neuropeptide Y Y1 Receptor (NPY1R) was significantly upregulated in aged granulosa cells. In vivo experiments confirmed that the NPY1R agonist [Leu^31^, Pro^34^]‐NPY induced estrous cycle disorder, inhibited follicular development, triggered reactive oxygen species (ROS) accumulation and mitochondrial structural damage in oocytes, thereby inducing ferroptosis, and ultimately leading to ovarian dysfunction and compromised oocyte quality; conversely, the NPY1R antagonist BIBO 3304 effectively reversed these phenotypes. Mechanistic studies demonstrated that NPY1R overexpression activates the p‐CREB signaling pathway, promotes the expression of pro‐inflammatory factor IL‐6 and inflammasome NLRP3, aggravates ferroptosis, mitochondrial dysfunction, and type I/III collagen imbalance, and ultimately accelerates ovarian aging. Collectively, this study clarifies that the NPY1R/CREB signaling axis participates in ovarian senescence by regulating inflammatory responses, and reveals its pivotal role in follicular development and oxidative stress. Targeting NPY1R is expected to be a potential therapeutic strategy for improving ovarian function and infertility in advanced‐age women.

## Graphical abstract

Aging‐associated upregulation of NPY1R drives p‐CREB activation, triggering inflammation, mitochondrial dysfunction, and extracellular matrix imbalance to promote ovarian senescence. Pharmacological inhibition of NPY1R with BIBO 3304 restores ovarian homeostasis, highlighting a therapeutic strategy for reproductive aging.

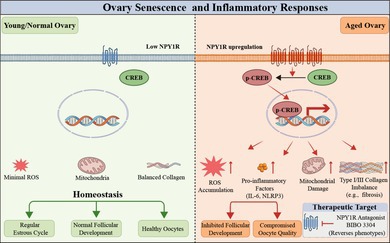

## 1 Introduction

Data from the World Health Organization show that the natural conception rate of women over 35 years old decreases by more than 50% compared with women aged 25–30 years old [1], and the success rate of in vitro fertilization (IVF) after 40 years old is less than 20%, accompanied by a significant increase in the risk of adverse pregnancy outcomes such as miscarriage and fetal chromosomal abnormalities [2]. This clinical situation not only imposes a heavy psychological and economic burden on families, but also poses a potential challenge to the sustainable development of the social population structure. With the popularization and application of assisted reproductive technology (ART), exploring effective approaches to improve ovarian function and gamete quality in elderly women, thereby elevating the clinical pregnancy rate, has become a core scientific issue that urgently needs to be solved in the field of reproductive medicine [3, 4].

As the core organ of the female reproductive system, the ovary's functional decline is the fundamental cause of reduced fertility in elderly women [5, 6]. This decline is primarily characterized by a marked depletion in oocyte quantity and a fundamental decline in oocyte quality. Among them, the dysfunction of ovarian granulosa cells (GCs) has been confirmed as a key initiating factor leading to impaired oocyte quality [7]. As the core supporting cells for follicular development, GCs construct and maintain the microenvironment for follicular development by secreting hormones, growth factors, and cytokines, and precisely regulate the processes of oocyte maturation, fertilization, and early embryonic development [8]. Studies have demonstrated that granulosa cell dysfunction is characterized by weakened proliferation, increased apoptosis rate, and imbalanced hormone secretion, which is precisely the important cytological basis for the formation of a chronic inflammatory microenvironment in the ovary [6].

The homeostasis of the ovarian microenvironment is a key prerequisite for normal follicular development and maintenance of oocyte quality, and the imbalance of inflammatory response plays a decisive driving role in the process of ovarian senescence [9]. Under physiological conditions, trace inflammatory factors secreted by granulosa cells participate in the fine regulation of follicular development; however, in the aged state, excessive accumulation of pro‐inflammatory factors results in a chronic low‐grade inflammatory state in the ovarian microenvironment [10, 11]. Clinical studies further confirm that the expression levels of pro‐inflammatory factors such as tumor necrosis factor‐α (TNF‐α), interleukin‐6 (IL‐6), and interleukin‐1β (IL‐1β) in ovarian granulosa cells and follicular fluid of women over 40 years old are significantly higher than those in young women [12], and the high expression of these inflammatory factors is significantly negatively correlated with oocyte maturation rate, fertilization rate, and clinical pregnancy rate [12, 13]. Excessive pro‐inflammatory factors can abnormally activate key inflammatory pathways such as nuclear factor kappa‐B (NF‐κB) [14], which not only accelerates granulosa cell apoptosis and interferes with oocyte maturation but also causes mitochondrial dysfunction in oocytes, thereby leading to a significant increase in oxidative stress levels [15, 16].

Neuropeptide Y (NPY) is a multifunctional neuropeptide widely distributed in the nervous system and peripheral tissues [17]. Its family comprises five receptors, among which Y1, Y2, Y4, and Y5 receptors are all G protein‐coupled receptors (GPCRs) [18], widely participating in the regulation of various physiological processes such as energy metabolism, stress response, and immune balance [19]. In patients with breast cancer complicated with osteoporosis, NPY1R is the most abundantly expressed peptide receptor [20]; studies have revealed that downregulation of NPY1R expression can promote osteogenic differentiation of bone marrow mesenchymal stem cells through the cAMP/PKA/CREB pathway, thereby improving bone mass [21]. In the female reproductive system, NPY not only participates in the regulation of uterine blood flow but also plays a regulatory role in vasoconstriction [22]; meanwhile, NPY can regulate the release of GnRH and participate in the formation of the LH surge [23, 24]. Clinical studies show that the expression level of NPY in the anterior vaginal wall mucosa of postmenopausal patients with pelvic organ prolapse is negatively correlated with the severity of the disease [25]. However, the specific function and mechanism of NPY1R in aged ovarian granulosa cells remain unclear. Notably, as an important member of the GPCRs family, activation of NPY1R can affect intracellular cyclic adenosine monophosphate (cAMP) levels by regulating the activity of adenylate cyclase (AC). As a classic second messenger, cAMP plays a key regulatory role in granulosa cell proliferation, hormone secretion, and follicular development [26, 27]. Studies have confirmed that gonadotropins (such as FSH) can promote granulosa cell maturation through the cAMP signaling pathway [26], and abnormalities in the cAMP signaling pathway are closely related to ovarian function decline [28].

In granulosa cells, the cAMP signaling pathway can participate in the dynamic regulation of the immune microenvironment by modulating the transcription of inflammation‐related genes [29]. It is hypothesized that the abnormal activation of the cAMP signaling pathway in aged granulosa cells may be an important upstream trigger for the imbalance of inflammatory responses. However, whether NPY1R regulates inflammatory responses in aged granulosa cells via modulating the cAMP signaling axis, as well as its specific downstream molecular mechanisms, remains experimentally unreported.

To address these research gaps, the present study aims to preliminarily elucidate the mechanism of senescence on ovarian function by using mouse models treated with NPY1R agonists or antagonists, combined with techniques such as HE staining of ovarian tissue, observation of mitochondrial ultrastructure and immunofluorescence. Follow‐up studies will focus on exploring whether precise intervention of the NPY1R‐CREB pathway can effectively attenuate senescence‐induced inflammatory responses in granulosa cells, thereby restoring ovarian microenvironment and function. This study integrally applies multidisciplinary approaches including cell biology, molecular biology and animal experiments to deeply analyze the regulatory network and molecular mechanisms of the NPY1R/CREB signaling axis in ovarian senescence. This work aims to provide novel theoretical insights and intervention targets for the prevention and treatment of ovarian dysfunction in advanced‐age women, and provide experimental basis and strategic support for improving female reproductive health.

## 2 Materials and Methods

### 2.1 Subjects of Human Primary Granulosa Cells

A total of 100 patients undergoing assisted reproductive technology treatment were enrolled and divided into the control group (*n* = 50) and advanced‐age group (*n* = 50) according to age and ovarian reserve function. Follicular fluid was collected from all patients for the isolation and purification of granulosa cells. This study was approved by the Ethics Committee of The First Affiliated Hospital of Zhengzhou University (Ethical Approval No.: 2025‐KY‐0760), and written informed consent was obtained from all patients. Inclusion criteria: (1) Control group: age < 35 years, anti‐Müllerian hormone (AMH) ≥ 1.1 ng/mL, normal ovarian reserve, basal antral follicle count (AFC) ≥ 5, body mass index (BMI) 18.5–23.9 kg/m^2^, and normal karyotype; (2) Aged group: age > 38 years, AFC ≥ 5, BMI 18.5–23.9 kg/m^2^, and normal karyotype. Exclusion criteria: (1) Specific ovarian diseases such as polycystic ovary syndrome and premature ovarian failure; (2) History of ovarian surgery; (3) Uterine malformation, intrauterine adhesion, uterine space‐occupying lesion, endometriosis, adenomyosis, or cervical incompetence; (4) Pelvic tuberculosis or hydrosalpinx; (5) Reproductive endocrine‐interfering diseases such as thyroid dysfunction and hyperprolactinemia; (6) Receiving radiotherapy or cytotoxic drug therapy in the past 3 months; (7) Family history of chromosomal abnormalities or monogenic genetic diseases.

### 2.2 Establishment and Feeding of Mouse Models

Two‐month‐old (young) and 10‐month‐old (aged) SPF‐grade female CD‐1 mice were purchased from Beijing Vital River Laboratory Animal Technology Co. Ltd. (Vital River, Beijing, China). Mice were housed in an SPF animal facility at a temperature of 22°C–25°C, humidity of 50%–60%, and a 12 h light/12 h dark cycle, with ad libitum access to standard chow and water. After 1 week of acclimatization, the experiment was conducted. Two‐month‐old mice were randomly divided into the normal diet group (Ctrl group) and NPY1R agonist diet group (Ctrl + [Leu^31^, Pro^34^]‐NPY group); 10‐month‐old mice were assigned to 12 months of age and then randomly divided into the normal diet group (Aged group) and NPY1R antagonist diet group (Aged + BIBO 3304 group). Mice in each group were allocated the corresponding diet daily with consistent food intake, body weight was monitored weekly, and mental and dietary status were observed until the experimental endpoint.

### 2.3 Purification and Culture of Human Primary Granulosa Cells

After thorough mixing, follicular fluid was centrifuged at 1500 rpm for 15 min at room temperature to remove impurities and red blood cells. The precipitate was resuspended in PBS and layered onto an equal volume of human lymphocyte separation medium (TBD Science, Tianjin, China), followed by centrifugation at 2000 rpm for 20 min for purification. The middle layer of granulosa cells was collected, washed twice with PBS, and seeded in DMEM/F‐12 medium containing 10% fetal bovine serum (FBS, Gibco, USA). Cells were cultured in a humidified incubator at 37°C, 5% CO_2_ incubator. Cell morphology and density were observed daily under an inverted microscope, and the culture medium was refreshed every 24 h. Subsequent experiments were performed when cell confluence reached 50%–60%.

### 2.4 Transcriptome Sequencing Analysis

Total RNA of granulosa cells was extracted using TRIzol reagent (Invitrogen, USA), and DNaseI (Takara, Beijing, China) was added to eliminate genomic DNA contamination. RNA integrity (RIN ≥ 7.0) was detected using a 2100 Bioanalyzer (Agilent Technologies, CA, USA), and RNA purity (A260/A280 = 1.8–2.0) was determined using an ND‐2000 Nucleic Acid Detector (NanoDrop Technologies, DE, USA). Qualified RNA was used to construct a library with the TruSep RNA Kit (Illumina, CA, USA), and high‐throughput sequencing (150 bp paired‐end reads) was performed on the Illumina HiSeq Xten/NovaSeq 6000 sequencing platform (Illumina). Differential gene expression analysis and functional analysis were performed subsequently.

### 2.5 Quantitative Real‐Time PCR (qPCR) Detection

Total RNA of cells or ovarian tissues was extracted using TRIzol reagent (Invitrogen, CA, USA), and cDNA was synthesized using a reverse transcription (RT) kit (Bio‐Rad Laboratories, CA, USA). Using cDNA as the template, quantitative real‐time PCR (qPCR) was performed on a 7500 Real‐Time PCR System (Bio‐Rad Laboratories) with SYBR Green Premix (Bio‐Rad). The thermal cycling conditions were as follows: pre‐denaturation at 95°C for 30 s; denaturation at 95°C for 5 s, annealing at 60°C for 30 s, for 40 cycles. The relative expression levels of target genes were calculated using the $2^{∆∆C_{t}}$ method, with GAPDH as the internal reference. Each sample was analyzed in triplicate. Primer sequences are shown in Table S1.

### 2.6 Western Blot Analysis

Cells were lysed on ice for 30 min with RIPA lysis buffer containing protease inhibitors (Sangon Biotech, Shanghai, China), and total protein was extracted by high‐speed refrigerated centrifugation at 12000 rpm for 15 min at 4°C. Protein concentration was determined by the BCA method. Equal amounts of protein were separated by SDS‐PAGE and transferred to a PVDF membrane, followed by blocking with 5% skimmed milk at room temperature for 1 h. Primary antibodies [GAPDH (Abcam, UK), NPY1R (Abcam, UK), CREB (Proteintech, China), p‐CREB (Proteintech, China), NLRP3 (CST, USA), IL‐6 (CST, USA), p‐ERK1/2 (CST, USA), ERK1/2 (CST, USA)] were added at a dilution ratio of 1:1000 and incubated overnight at 4°C. After washing with TBST, mouse/rabbit secondary antibodies (Abcam, UK) were added at a dilution ratio of 1:5000 and incubated at room temperature for 1 h. Protein bands were detected using an enhanced chemiluminescence detection system (Bio‐Rad Laboratories), and quantitative analysis was performed using ImageJ software, with GAPDH serving as the internal reference.

### 2.7 Collection and Counting of Mouse Oocytes

Mice were intraperitoneally injected with pregnant mare serum gonadotropin (PMSG, 100 IU/mL, 0.1 mL) to induce superovulation, and human chorionic gonadotropin (hCG, 100 IU/mL, 0.1 mL) was injected 48 h later. Mice were euthanized 14 h after hCG injection, and the ampulla of the oviducts was dissected. Cumulus‐oocyte complexes (COCs) were retrieved in G‐MOPS buffer (Vitrolife, Sweden) and treated with hyaluronidase to remove cumulus cells. After washing with KSOM, the number of oocytes was counted under an inverted light microscope.

### 2.8 Detection of Oocyte Mitochondrial Membrane Potential (MMP)

Oocytes were incubated with 2 μM JC‐1 reagent (Beyotime, Shanghai, China) in a 37°C, 5% CO_2_ incubator for 30 min. After washing with PBS, images were captured using a Carl Zeiss laser confocal microscope (Carl Zeiss, Oberkochen, Germany) (excitation wavelength: 488 nm, emission wavelengths: 525 and 590 nm). The ratio of red to green fluorescence intensity was quantified using ImageJ software.

### 2.9 Detection of Oocyte Reactive Oxygen Species (ROS)

Oocytes were incubated with 10 μM carboxy‐2′,7′‐dichlorodihydrofluorescein diacetate (Beyotime) in a 37°C, 5% CO_2_ incubator for 30 min. After washing with PBS, fluorescence images were captured using a Carl Zeiss laser confocal microscope (excitation wavelength: 488 nm, emission wavelength: 525 nm). Fluorescence intensity was quantitatively analyzed with ImageJ software.

### 2.10 Cell Transfection

Cells were seeded in a 6‐well plate and cultured until they reached 70% confluence, at which point the medium was replaced with serum‐free RPMI 1640 medium. Transfection complexes were prepared using Opti‐MEM, and Lipofectamine 3000 (Thermo Fisher Scientific, USA) and siRNA (GenePharma, Shanghai, China) were added. After 6 h of incubation, the medium was replaced with complete growth medium, and cells were cultured for an additional 48 h for subsequent detection.

### 2.11 Cell Proliferation and Toxicity Assay (CCK‐8 Method)

KGN cells were seeded in 96‐well plates. After adherence, cells were treated with various concentrations of drugs and cultured for 48 h. CCK‐8 working solution (10 μL CCK8 reagent + 100 μL DMEM/F‐12 medium) was added, followed by incubation for 120 min. The absorbance (OD value) at 450 nm was measured using a microplate reader.

### 2.12 HE Staining and Follicle Counting of Ovarian Tissues

Ovarian tissues were fixed overnight in 4% paraformaldehyde at 4°C, embedded in paraffin using a paraffin embedding machine, and sectioned into 5 μm slices with a microtome. After dewaxing and rehydration, sections were stained with an HE staining kit, followed by gradient dehydration, transparency, and mounting with neutral balsam. Tissue morphology was observed under a light microscope; the number of follicles at all levels was counted, and the average number of follicles was calculated.

### 2.13 Detection of Mouse Estrous Cycle

Vaginal smears were collected at a fixed time daily, air‐dried naturally, fixed in 95% ethanol, and stained with an HE staining kit. Cell morphology was observed under a stereomicroscope to determine the estrous cycle stage (proestrus, estrus, metestrus, diestrus). Continuous recording was performed for 14 days to analyze cycle disorders.

### 2.14 Observation of Ovarian Tissue Mitochondrial Ultrastructure

Ovarian tissues were cut into approximately 1 mm^3^ pieces, fixed with 4% paraformaldehyde, and then fixed in 1% OsO_4_ fixative at room temperature in the dark for 2 h. After gradient dehydration, acetone transparency, resin embedding, and baking polymerization at 60°C, tissues were cut into 50–80 nm ultrathin sections with an ultramicrotome. Sections were picked up with carbon‐coated copper grids, stained with uranium and lead, and observed and photographed under a transmission electron microscope (TEM) to analyze mitochondrial ultrastructure.

### 2.15 Immunofluorescence Staining

Ovarian paraffin sections were dewaxed in environmentally friendly dewaxing solution I, II, and III (Servicebio, China) for 10 min each, followed by dehydration in anhydrous ethanol I, II, and III for 5 min each. After rinsing with tap water, antigen retrieval was performed in a 90°C water bath for 30 min. After antigen retrieval, a hydrophobic circle was drawn around the sample using a histochemical pen (Servicebio, China), and 3% BSA blocking solution (Servicebio) was added for blocking solution at room temperature for 30 min to block nonspecific binding. After blocking, corresponding primary antibodies were added, and sections were placed flat in a wet box and incubated overnight at 4°C. Primary antibodies and dilution ratios were as follows: p‐CREB (Servicebio, 1:700), CREB (Servicebio, 1:500), IL‐6 (Servicebio, 1:200), NLRP3 (Servicebio, 1:200). On the next day, primary antibodies were discarded, sections were washed with PBS, and goat anti‐rabbit secondary antibody (Servicebio, 1:300) was added for incubation at room temperature in the dark for 50 min. After washing with PBS three times, DAPI staining solution (Servicebio) was added and incubated at room temperature in the dark for 10 min. Autofluorescence quencher B (Servicebio) was then added and incubated for 5 min, followed by rinsing with running water for 10 min to remove residual dye. Finally, sections were mounted with anti‐fade mounting medium (Servicebio), observed under a microscope, and images were acquired.

### 2.16 PSR Staining of Ovarian Tissues (Fibrosis Detection)

Ovarian paraffin sections were dewaxed in environmentally friendly dewaxing solution I, II, and III for 10 min each, dehydration in anhydrous ethanol I and II for 5 min each, and 75% ethanol for 5 min, then rinsed with tap water until transparent. Sections were immersed in modified Picrosirius Red (PSR) staining solution A (Servicebio) and incubated in a 65°C oven for 30 min; sections were then immersed in solution B for 2 min and solution C for 1 h. After staining, sections were rinsed with tap water to remove excess dye, followed by rapid rinsing in three cylinders of anhydrous ethanol for 3–5 s each, transparency in xylene (Lingfenng, Shanghai) for 5 min, and mounted with neutral balsam (Sinopharm, China). Sections were observed under a polarized microscope and images were collected. The percentage of fibrotic area was quantified using ImageJ software.

### 2.17 Statistical Analysis

SPSS 22.0 software was used for statistical analysis. Measurement data were expressed as “mean ± standard deviation (x±s),” and comparisons between two groups were performed using the *t*‐test or Mann–Whitney *U* test; enumeration data were expressed as rate (%), and group comparisons were analyzed using the χ^2^ test. *p* < 0.05 was considered statistically significant. All experiments were independently repeated at least three times.

## 3 Results

### 3.1 NPY1R Is Upregulated in Aged Ovarian Granulosa Cells and Is Highly Correlated With Senescence‐Related Pathways

**FIGURE 1:**
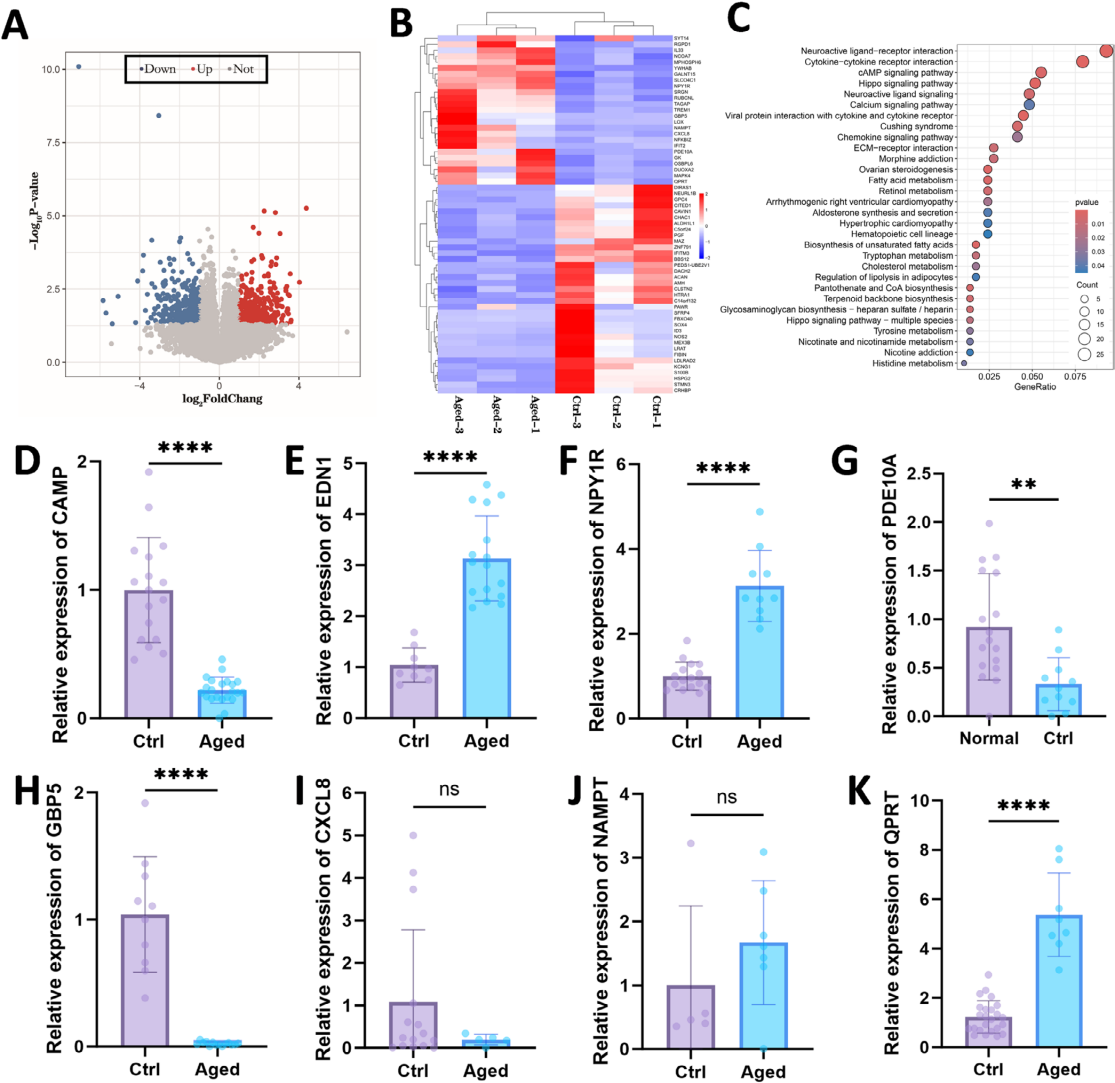
NPY1R is upregulated in aged ovarian granulosa cells and correlated with senescence‐related pathways. (A) Volcano plot of differentially expressed genes. With |log_2_FC| > 1 and adjusted *p* value (*p*adj) < 0.05 as screening thresholds, a total of 1704 DEGs were identified, including 844 upregulated genes (red dots) and 860 downregulated genes (blue dots). (B) Heatmap of DEG expression profiles. Clustering of the top 30 genes with the most significant expression differences between the two groups is shown, with distinct separation patterns between upregulated and downregulated genes. (C) KEGG pathway enrichment analysis of DEGs. Major signaling pathways significantly enriched in DEGs of aged ovarian granulosa cells are displayed. (D–K) qPCR validation of DEGs. The relative mRNA expression levels of several candidate DEGs in the cAMP signaling pathway (including in panel F) were detected in primary granulosa cells from patients with normal ovarian function (Ctrl) and advanced‐age patients (Aged) via qPCR. Data are presented as mean ± standard deviation (Mean ± SD). **p < 0.01, ****p < 0.0001; ns, no statistically significant difference.

To elucidate the molecular mechanisms underlying ovarian aging in advanced‐age women, ovarian granulosa cells were collected from patients with normal ovarian function (control group) and advanced‐age patients of age‐stratified elderly patient (aged group) for transcriptome sequencing (RNA‐seq) analysis. Differential expression analysis revealed 1704 differentially expressed genes (DEGs) in ovarian granulosa cells of the aged group compared with the control group (Ctrl vs. Aged), including 844 significantly upregulated genes and 860 significantly downregulated genes (Figure 1A). In‐depth analysis of DEGs showed that multiple significantly upregulated genes (including *PDE10A*, *QPRT*, *NPY1R*, and *GBP5*) and significantly downregulated genes (including *EDN1*, *NAMPT*, and *AMH*) have been reported to be closely associated with normal follicular development, hormone secretion, and fine‐tuned regulation of reproductive function in the ovary (Figure 1B). To uncover the potential biological processes involving these DEGs, KEGG pathway enrichment analysis was further performed. The results demonstrated that the *neuroactive ligand‐receptor interaction*, *cytokine‐cytokine receptor interaction*, *cAMP signaling pathway*, and *Hippo signaling pathway* exhibited the most significant enrichment trends among the DEG sets (Figure 1C). Notably, these pathways have been widely proven to play pivotal regulatory roles in follicular growth, development, atresia, and maintenance of ovarian homeostasis, and are closely linked to the reproductive aging process in women. Among them, the *cAMP signaling pathway* is directly related to ovarian reserve function and age‐related functional decline. To verify the reliability of transcriptome analysis and clarify the specific regulatory roles of cAMP signaling pathway‐related genes in aged ovarian dysfunction, quantitative real‐time polymerase chain reaction (qPCR) was used to detect the mRNA expression levels of several key cAMP pathway regulatory genes in granulosa cells from clinical samples. Validation results (Figure 1D–K) showed that *NPY1R* expression was significantly elevated in granulosa cells of elderly patients compared with the normal control group (3.15‐fold; *p* < 0.0001), which was fully consistent with the transcriptome sequencing data (Figure 1F). This finding suggests that *NPY1R* may act as a key upstream regulator of the cAMP signaling pathway, and its aberrant high expression may be closely related to dysfunction and senescence of aged ovarian granulosa cells.

### 3.2 NPY1R Agonist Induces Ovarian Dysfunction and Oocyte Quality Decline in Young Mice

**FIGURE 2:**
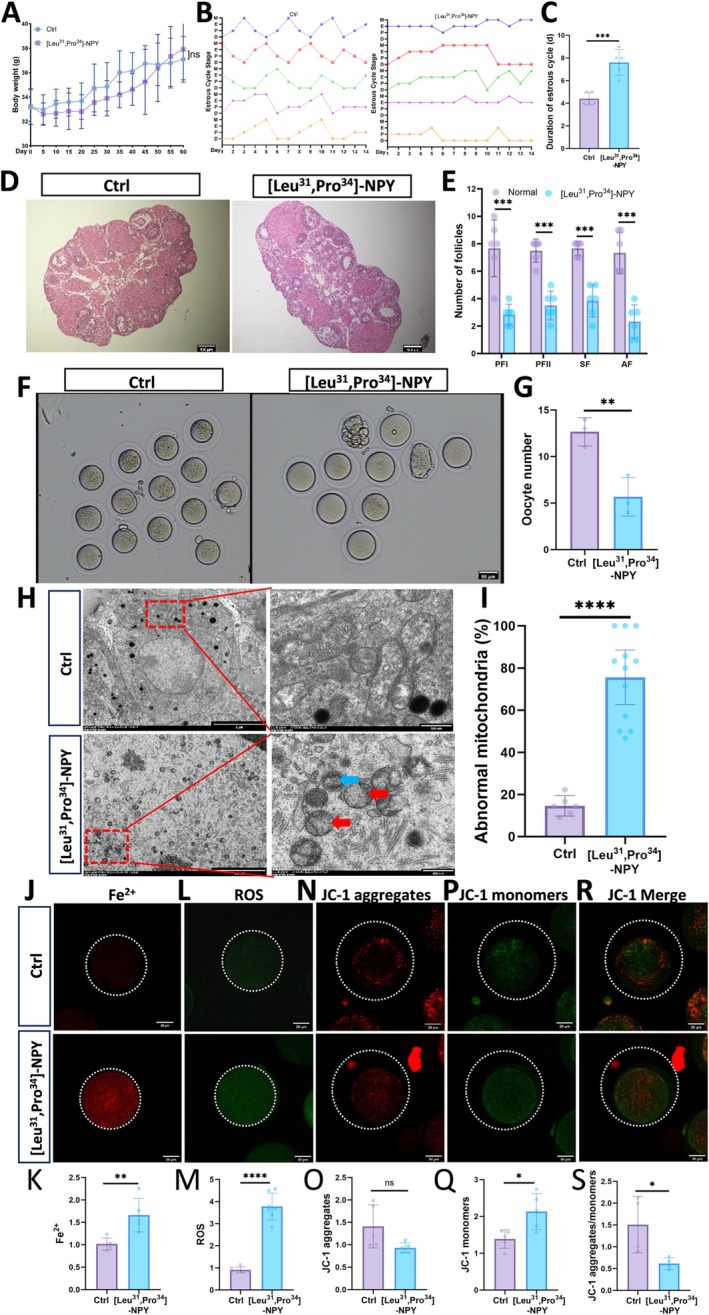
NPY1R agonist treatment impairs ovarian function and oocyte quality in young mice. (A) Body weight monitoring. Eight‐week‐old female CD1 mice were randomly divided into a normal diet control group (Ctrl) or an agonist diet group ([Leu^31^, Pro^34^]‐NPY), and body weight was monitored every 5 days. (B, C) Estrous cycle analysis. Starting from the 6th week of the experiment, vaginal smears were collected daily from 8:00 to 9:00 for 14 consecutive days, stained with HE, and observed under a light microscope to determine estrous cycle stages (P: Proestrus; E: Estrus; M: Metestrus; D: Diestrus); estrous cycle duration was statistically analyzed. (D, E) Ovarian histology and follicle counting. At the end of the experiment, ovarian tissues were harvested, fixed with 4% paraformaldehyde, and stained with HE to observe follicular development; the number of follicles at each stage was counted (PFI: Primordial follicle; PFII: Primary follicle; SF: Secondary follicle; AF: Antral follicle). Scale bar = 200 μm. (F, G) Statistics of oocyte yield after superovulation. After superovulation, cumulus‐oocyte complexes (COCs) were collected from the ampullae of the two groups, denuded of cumulus cells with hyaluronidase, and oocytes were counted under an inverted microscope. Scale bar = 50 μm. (H, I) Ultrastructural analysis of oocyte mitochondria. Ovarian tissues were cut into 1 × 1 × 1 mm^3^ pieces, preserved in electron microscopy fixative, and mitochondrial morphology was observed via TEM (red arrow: Cristae loss; blue arrow: Swelling and rupture). Scale bars: Left 5 μm, right 500 nm. (J–S) Detection of oocyte oxidative stress and mitochondrial function. Oocytes were incubated with Fe^2+^, ROS, and JC‐1 staining solutions respectively, photographed under a laser confocal microscope, and fluorescence intensity was quantitatively analyzed (Fe^2+^: J, K; ROS: L, M; JC‐1 Red/Green ratio: N–S). Scale bar = 20 μm. *p < 0.05, **p < 0.01, ***p < 0.001, ****p < 0.0001; ns, no statistically significant difference.

To investigate the effects of NPY1R agonist on the mouse reproductive system, 8‐week‐old female CD1 mice were administered the NPY1R agonist ([Leu^31^, Pro^34^]‐NPY) to evaluate its impacts on ovarian function and oocyte quality. Animals were randomly divided into a normal diet control group (Ctrl) and an agonist‐treated group ([Leu^31^, Pro^34^]‐NPY), with an 8‐week intervention period. Body weight was monitored every 5 days throughout the experiment, and no significant difference was observed between the two groups (*p* > 0.05) (Figure 2A), indicating that agonist treatment had no significant effect on the overall metabolism of mice. To assess reproductive endocrine function, vaginal smears were collected and stained for 14 consecutive days starting from the 6th week of the experiment. The results showed that the estrous cycle of control mice (4–5 days) was significantly shorter than that of the agonist‐treated group (6–10 days, *p* < 0.05), and all stages of the estrous cycle (proestrus P, estrus E, metestrus M, diestrus D) were prolonged to varying degrees in the agonist‐treated group (Figure 2B,C). This suggests that NPY1R agonist may disrupt hypothalamic–pituitary‐ovarian axis function, leading to estrous cycle disorders.

Further hematoxylin–eosin (HE) staining of ovarian tissue sections revealed that the number of follicles at all stages (primordial follicles PFI, primary follicles PFII, secondary follicles SF, antral follicles AF) was significantly lower in the agonist‐treated group than in the control group (*p* < 0.001) (Figure 2D,E), indicating that NPY1R agonist may reduce ovarian reserve by inhibiting follicular development or accelerating follicular atresia. To explore the impact on oocytes, oocytes retrieved after superovulation were counted, and the number of oocytes obtained was significantly lower in the agonist‐treated group than in the control group (*p* < 0.001) (Figure 2F,G), consistent with histological findings and further confirming that the agonist may reduce oocyte quantity by impairing follicular recruitment or promoting follicular apoptosis.

Mitochondrial function is a critical determinant of oocyte quality. To clarify the effects of NPY1R agonist on the mitochondrial ultrastructure of granulosa cells, transmission electron microscopy (TEM) observation showed that mitochondria in control oocytes were regularly shaped (long elliptical or short rod‐shaped), with tightly arranged and clear cristae; in contrast, some mitochondria in the agonist‐treated group exhibited swelling, deformation, and cristae rupture, blurring or even loss (Figure 2H). Statistical analysis of abnormal mitochondria showed that the proportion was significantly higher in the agonist‐treated group than in the control group (*p* < 0.0001) (Figure 2I), suggesting that NPY1R agonist may reduce oocyte quality by damaging mitochondrial structure and function. Subsequently, key indicators of oocyte oxidative stress were analyzed. The results showed that Fe^2+^ fluorescence intensity in oocytes was significantly higher in the agonist‐treated group than in the control group (*p* < 0.01) (Figure 2J,K), indicating that NPY1R agonist may induce oxidative stress injury by promoting iron ion accumulation. Further detection via reactive oxygen species (ROS) and JC‐1 staining revealed that ROS levels were significantly increased (*p* < 0.0001) (Figure 2L,M) and the JC‐1 Red/Green ratio (reflecting mitochondrial membrane potential) was significantly decreased (*p* < 0.05) (Figure 2N–S) in oocytes of the agonist‐treated group. This indicates reduced mitochondrial membrane potential and elevated oxidative stress, which may cause abnormal energy metabolism in oocytes and impair their developmental potential.

### 3.3 NPY1R Antagonist BIBO 3304 Improves Ovarian Function and Enhances Oocyte Quality in Aged Mice

**FIGURE 3:**
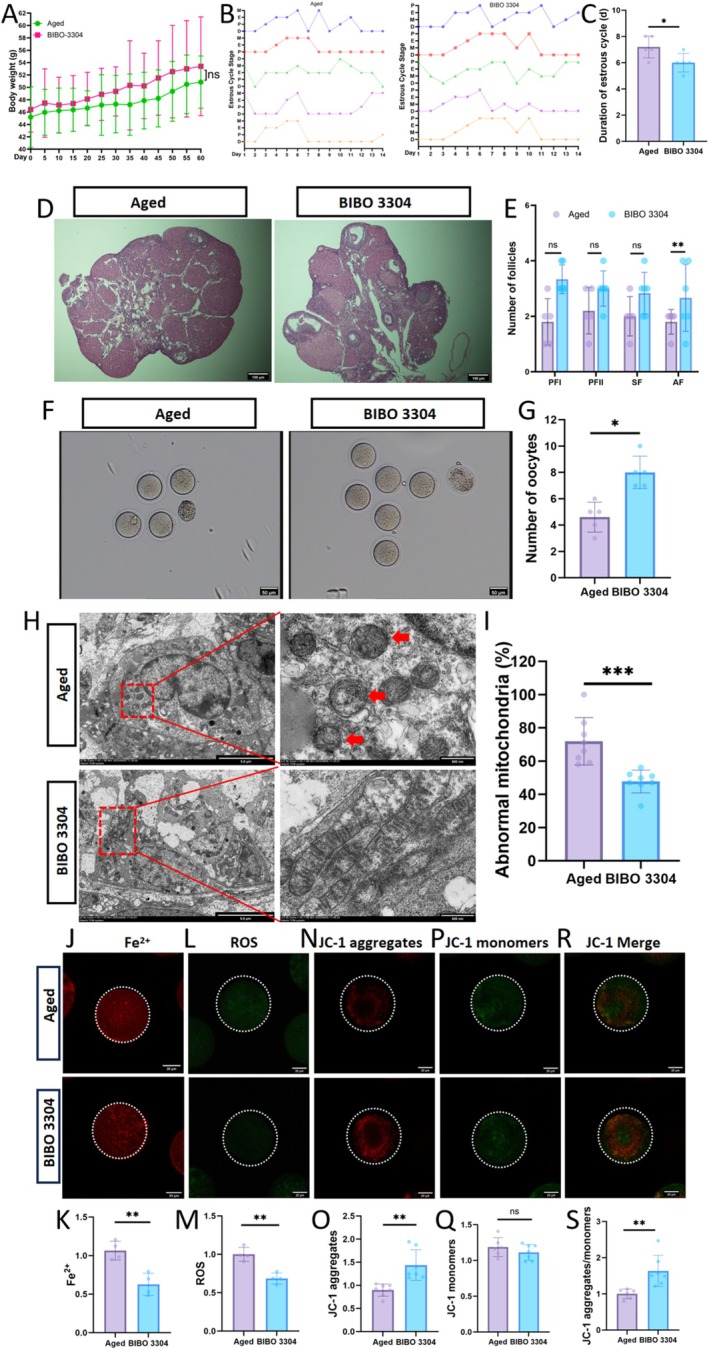
NPY1R antagonist BIBO 3304 improves ovarian function and oocyte quality in aged mice. (A) Body weight monitoring. Twelve‐month‐old female CD1 mice were randomly divided into a normal diet group (Aged) or an NPY1R antagonist diet group (BIBO 3304), and body weight was monitored every 5 days. (B, C) Estrous cycle analysis. Starting from the 6th week of the experiment, vaginal smears were collected daily for 14 consecutive days, stained with HE, and observed under a light microscope to determine estrous cycle stages; estrous cycle duration was statistically analyzed. (D, E) Ovarian histology and follicle counting. At the end of the experiment, ovarian tissues were harvested, fixed with 4% paraformaldehyde, and stained with HE to observe follicular development; the number of follicles at each stage was counted. Scale bar = 200 μm. (F, G) Statistics of oocyte yield after superovulation. After superovulation, COCs were collected from the ampullae of the two groups, denuded of cumulus cells with hyaluronidase, and oocytes were counted under an inverted microscope. Scale bar = 50 μm. (H, I) Ultrastructural analysis of oocyte mitochondria. Ovarian tissues were cut into 1 × 1 × 1 mm^3^ pieces, preserved in electron microscopy fixative, and mitochondrial morphology was observed via TEM (red arrow: Swelling and rupture). Scale bars: Left 5 μm, right 500 nm. (J–S) Detection of oocyte oxidative stress and mitochondrial function. Oocytes were incubated with Fe^2+^, ROS, and JC‐1 staining solutions respectively, photographed under a laser confocal microscope, and fluorescence intensity was quantitatively analyzed (Fe^2+^: J, K; ROS: L, M; JC‐1 Red/Green ratio: N–S). Scale bar = 20 μm. Data are presented as mean ± SD. *p < 0.05, **p < 0.01, ***p < 0.001; ns, no statistically significant difference.

To explore the regulatory effect of NPY1R antagonist on ovarian function and oocyte quality in aged female mice, 12‐month‐old female CD1 mice were randomly divided into a normal diet group (Aged) and an NPY1R antagonist BIBO 3304 diet group (BIBO 3304), with an 8‐week intervention period. Body weight was monitored every 5 days during the experiment, and no significant difference was detected between the two groups (*p* > 0.05) (Figure 3A), indicating that BIBO 3304 had no significant impact on the overall metabolism of mice.

To evaluate reproductive endocrine function, vaginal smears were collected and stained for 14 consecutive days starting from the 6th week of the experiment. The results showed that the estrous cycle of aged mice was significantly longer than that of the BIBO 3304 group (*p* < 0.05) (Figure 3B,C), demonstrating that NPY1R antagonist can ameliorate estrous cycle disorders in aged mice. Further HE staining of ovarian tissue sections revealed no significant differences in the number of primordial follicles (PFI), primary follicles (PFII), and secondary follicles (SF) between the aged group and BIBO 3304 group (*p* > 0.05), but the number of antral follicles (AF) was significantly increased in the BIBO 3304 group compared with the aged group (*p* < 0.01) (Figure 3D,E). This result suggests that NPY1R antagonist may promote antral follicle development. To verify this hypothesis, oocytes retrieved after superovulation were counted, and the oocyte yield was significantly higher in the BIBO 3304‐treated group than in the aged control group (*p* < 0.05) (Figure 3F,G), consistent with histological findings.

Mitochondrial function is a key indicator of oocyte quality. TEM observation showed that mitochondria in oocytes of the aged group generally exhibited abnormalities such as swelling, cristae rupture, and blurred membrane structures, while mitochondrial morphology in the BIBO 3304 group was regular with tightly arranged cristae (Figure 3H). Statistical analysis of abnormal mitochondria showed that the number of abnormal mitochondria was significantly reduced in the BIBO 3304 group compared with the aged group (*p* < 0.001) (Figure 3I), suggesting that the NPY1R antagonist may protect mitochondrial ultrastructure by inhibiting oxidative stress, thereby improving oocyte function. To clarify the improving effect of BIBO 3304 on oocyte quality, intracellular Fe^2+^ levels, ROS levels, and mitochondrial membrane potential (JC‐1 Red/Green ratio) were detected. The results showed that Fe^2+^ and ROS levels in oocytes were significantly higher in the aged group than in the BIBO 3304 group (*p* < 0.01) (Figure 3J–M), and the JC‐1 Red/Green ratio was significantly increased in the latter (*p* < 0.01) (Figure 3N–S), indicating that the NPY1R antagonist can reduce oxidative stress and restore mitochondrial membrane potential, thereby improving oocyte energy metabolism.

### 3.4 NPY1R Activity Intervention Regulates the Expression of Inflammatory Factors IL‐6 and NLRP3 in Ovarian Granulosa Cells

**FIGURE 4:**
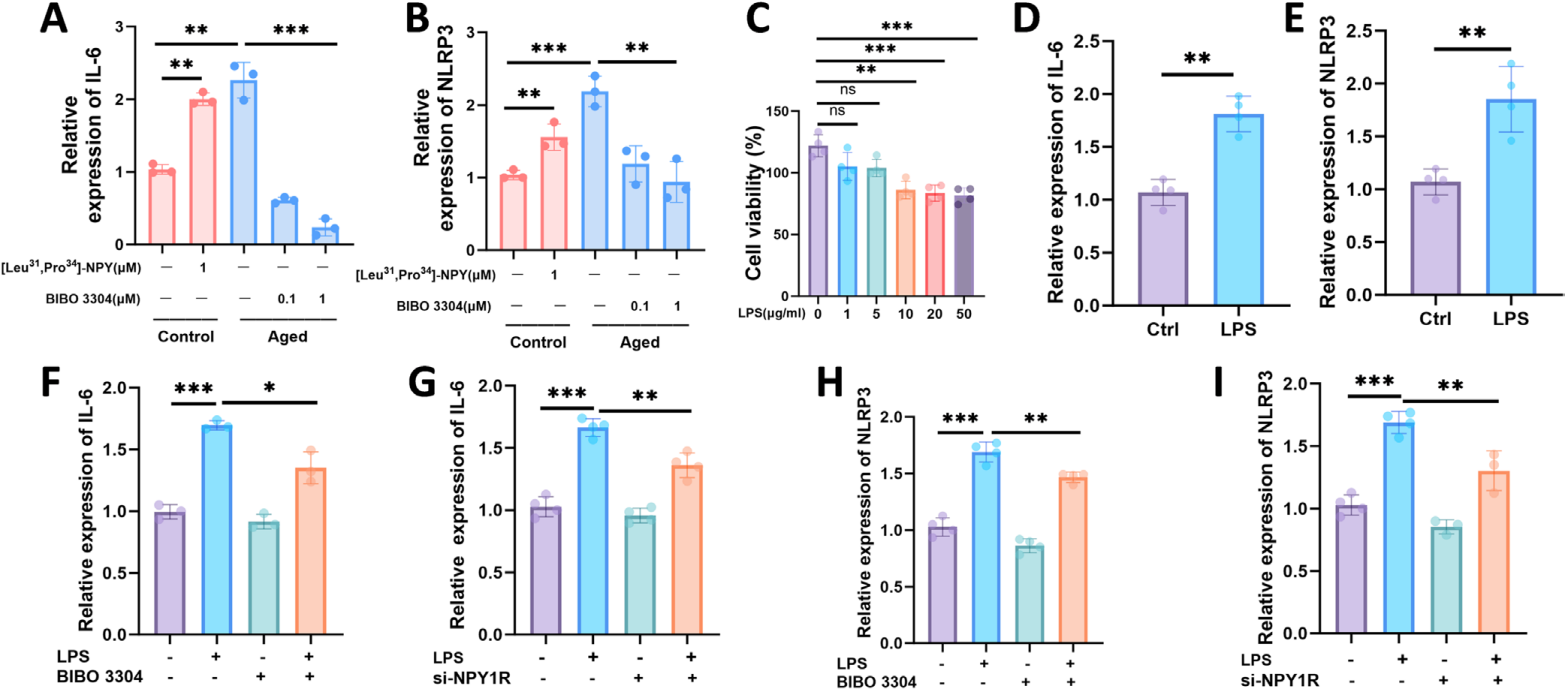
Regulation of inflammatory factors IL‐6 and NLRP3 expression in granulosa cells by NPY1R activity intervention. (A, B) Effects of NPY1R agonist and antagonist on inflammatory factor expression. Primary granulosa cells were isolated from normal (Ctrl) and aged (Aged) patients and treated with NPY1R agonist ([Leu^31^, Pro^34^]‐NPY, 1 μM) or antagonist (BIBO 3304, 0.1 μM and 1 μM) for 48 h after adherence; mRNA expression levels of *IL‐6* (A) and *NLRP3* (B) were detected via qPCR. (C) Effect of LPS on KGN cell viability. KGN cells were seeded in 96‐well plates at a density of 4 × 10^4^ cells/well, treated with different concentrations of LPS for 48 h, and cell viability was assessed by measuring absorbance (OD value) at 450 nm using the CCK‐8 assay, and untreated control cell viability is set to 100%. (D, E) LPS‐induced expression of inflammatory factors in granulosa cells. Normal primary granulosa cells were stimulated with LPS (10 μg/mL) for 6 h, and mRNA expression levels of *IL‐6* (D) and *NLRP3* (E) were detected via qPCR. (F–I) Intervention of NPY1R inhibits LPS‐induced inflammatory responses. Normal primary granulosa cells were treated with NPY1R antagonist BIBO 3304 (F, H) or transfected with siRNA to knockdown *NPY1R* (G, I) simultaneously with LPS (10 μg/mL) treatment; the inhibitory effects on LPS‐induced upregulation of *IL‐6* (F, G) and *NLRP3* (H, I) expression were detected via qPCR. Data are presented as mean ± SD. *p < 0.05, **p < 0.01, ***p < 0.001; ns, no statistically significant difference.

Previous studies have reported elevated expression of inflammatory factors in ovarian tissues of elderly women. Consistent with this, the present study found that the mRNA expression levels of inflammatory factors *IL‐6* and *NLRP3* were significantly higher in primary ovarian granulosa cells isolated from advanced‐age patients than in the normal control group (*p* < 0.01) (Figure 4A,B). Combined with the previous finding that *NPY1R* was significantly upregulated in granulosa cells of advanced‐age patients, we further investigated whether NPY1R activity directly regulates the expression of these inflammatory factors.

Treatment of primary granulosa cells from normal patients with the specific NPY1R agonist [Leu^31^, Pro^34^]‐NPY significantly induced the upregulation of *IL‐6* and *NLRP3* expression (*p* < 0.01). In contrast, treatment of primary granulosa cells from advanced‐age patients with the NPY1R antagonist BIBO 3304 significantly inhibited the expression of *IL‐6* and *NLRP3* (*p* < 0.05) (Figure 4A,B). These results indicate that pharmacological intervention of NPY1R activity can bidirectionally regulate the expression of key inflammatory factors in granulosa cells.

Lipopolysaccharide (LPS) is a classic reagent for inducing cellular inflammatory responses. To establish an in vitro inflammation model, human granulosa cell line KGN cells were treated with different concentrations of LPS (0, 1, 5, 10, 20, 50 μg/mL) for 48 h, and cell viability was detected via the CCK‐8 assay. The results showed that LPS concentrations ≥ 5 μg/mL significantly inhibited cell viability (*p* < 0.01), with the most significant inhibitory effect observed at 10 μg/mL (*p* < 0.001) (Figure 4C). Therefore, 10 μg/mL LPS was selected as the induction concentration for subsequent experiments. At this concentration, LPS treatment of human primary granulosa cells for 6 h effectively and significantly upregulated the mRNA expression levels of *IL‐6* and *NLRP3* (*p* < 0.01) (Figure 4D,E), successfully constructing a granulosa cell inflammation model.

Based on the above finding that *NPY1R* and inflammatory factors were synchronously upregulated in aged granulosa cells, we further explored whether NPY1R directly participates in regulating inflammatory factor expression. First, *NPY1R* expression was successfully modulated in cell models via adenovirus‐mediated overexpression and siRNA‐mediated knockdown (Figure S1). Subsequently, NPY1R function was intervened using pharmacological inhibition (BIBO 3304) and gene silencing strategies in the LPS‐induced inflammation model. The results showed that the NPY1R antagonist BIBO 3304 significantly inhibited LPS‐induced upregulation of *IL‐6* expression (*p* < 0.05) (Figure 4F). Similarly, siRNA‐mediated knockdown of *NPY1R* also significantly suppressed LPS‐induced elevation of *IL‐6* expression (*p* < 0.01) (Figure 4G). For another key inflammatory factor NLRP3, both NPY1R antagonist BIBO 3304 (Figure 4H) and gene knockdown (Figure 4I) significantly reversed LPS‐induced upregulation of *NLRP3* expression (*p* < 0.01). These results consistently confirm that *NPY1R* is a key molecule regulating the expression of inflammatory factors IL‐6 and NLRP3 in granulosa cells.

### 3.5 NPY1R Promotes Granulosa Cell Inflammatory Response by Activating the CREB Phosphorylation Pathway

**FIGURE 5:**
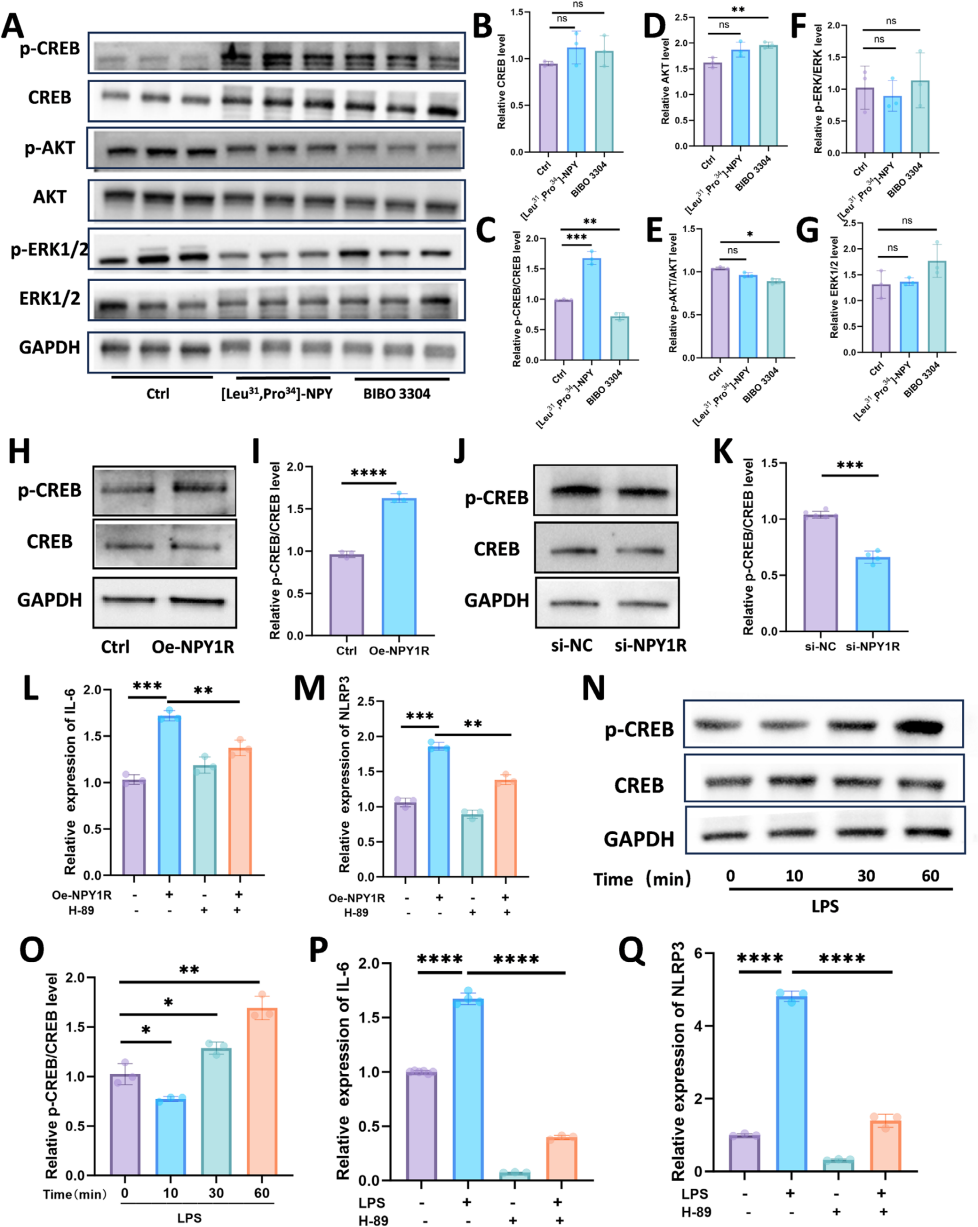
NPY1R regulates granulosa cell inflammatory response via the CREB phosphorylation pathway. (A–G) Effects of NPY1R agonist/antagonist on signaling pathway proteins. Human primary granulosa cells were treated with [Leu^31^, Pro^34^]‐NPY (1 μM) or BIBO 3304 (1 μM) for 48 h; protein expression levels of p‐CREB, CREB, p‐AKT, AKT, p‐ERK1/2, and ERK1/2 were detected via Western Blot. GAPDH was used as the internal reference protein. Gray value analysis was performed on Western Blot bands, and histograms represent the gray value ratio of phosphorylated protein to total protein (B–G). (H–I) Effect of NPY1R overexpression on CREB phosphorylation. KGN cells were treated with adenovirus‐mediated NPY1R overexpression (Oe‐NPY1R) for 48 h; p‐CREB and CREB protein levels were detected (H) and gray value quantitative analysis was performed (I). (J–K) Effect of NPY1R knockdown on CREB phosphorylation. Human primary granulosa cells were transfected with siRNA to knockdown *NPY1R* (si‐NPY1R) for 48 h; p‐CREB and CREB protein levels were detected (J) and gray value quantitative analysis was performed (K). (L–M) Inhibitory effect of H‐89 on NPY1R overexpression‐induced inflammation. Primary granulosa cells were treated with Oe‐NPY1R and pretreated with CREB phosphorylation inhibitor H‐89 (10 μM) for 1 h simultaneously; mRNA expression levels of *IL‐6* and *NLRP3* were detected via qPCR after 48 h (L, M). (N–O) Temporal analysis of LPS‐induced CREB phosphorylation. KGN cells were treated with LPS (10 μg/mL) for 0, 10, 30, and 60 min; the 60‐min time point was used for subsequent inhibition experiments. Total protein was extracted to detect p‐CREB and CREB levels (N), and gray value quantitative analysis was performed (O). (P–Q) Inhibitory effect of H‐89 on LPS‐induced inflammation. Primary granulosa cells were pretreated with H‐89 (10 μM) for 1 h, then treated with LPS (10 μg/mL) for 60 min; mRNA expression levels of *IL‐6* and *NLRP3* were detected via qPCR (P, Q). Data are presented as mean ± SD. *p < 0.05, **p < 0.01, ***p < 0.001, ****p < 0.0001; ns, no statistically significant difference.

To investigate the regulatory mechanism of NPY1R on granulosa cell inflammatory responses, human primary granulosa cells were treated with NPY1R agonist [Leu^31^, Pro^34^]‐NPY and antagonist BIBO 3304 respectively, and the phosphorylation levels of related signaling pathway proteins were detected. Western Blot results revealed that the p‐CREB/CREB ratio was significantly upregulated in the [Leu^31^, Pro^34^]‐NPY‐treated group and significantly downregulated in the BIBO 3304‐treated group (Figure 5A–F). In contrast, the phosphorylation levels of p‐AKT/AKT and p‐ERK1/2/ERK1/2 demonstrated no significant differences among groups. These results indicate that activation or inhibition of NPY1R specifically regulates the CREB phosphorylation pathway without significantly affecting the AKT or ERK1/2 pathways.

To further verify the direct regulatory role of NPY1R on CREB phosphorylation, *NPY1R* was overexpressed (Oe‐NPY1R) in normal primary granulosa cells. After 48 h, western blot analysis showed that the p‐CREB/CREB level was significantly increased in the overexpression group (Figure 5H,I), confirming that NPY1R overexpression serves as a critical upstream regulator of the CREB phosphorylation pathway. Conversely, knockdown of *NPY1R* expression (si‐NPY1R) in primary granulosa cells from advanced‐age patients significantly reduced the p‐CREB/CREB level (Figure 5J,K), further supporting the positive regulatory role of NPY1R on CREB phosphorylation.

Previous experiments confirmed that NPY1R overexpression induces upregulation of *IL‐6* and *NLRP3* mRNA expression. To clarify the role of CREB phosphorylation in this process, cells were co‐treated with the CREB phosphorylation inhibitor H‐89. qPCR results showed that H‐89 significantly reversed Oe‐NPY1R‐induced upregulation of *IL‐6* and *NLRP3* expression (*p* < 0.01) (Figure 5L,M), indicating that CREB phosphorylation is a key downstream mechanism by which NPY1R promotes inflammatory responses.

To explore the dynamic changes of the NPY1R/CREB pathway in inflammatory responses, human granulosa cell line KGN cells were treated with LPS, and the temporal changes in p‐CREB phosphorylation level were detected. Western Blot analysis showed that p‐CREB content and the p‐CREB/CREB ratio were significantly increased after 60 min of LPS treatment (*p* < 0.01) (Figure 5N,O), suggesting that this time point is the optimal window for detecting LPS‐induced CREB phosphorylation. In addition, H‐89 pretreatment significantly inhibited LPS‐induced upregulation of *IL‐6* and *NLRP3* expression (*p* < 0.0001) (Figure 5P,Q), further confirming that CREB phosphorylation is a core link in NPY1R‐mediated LPS inflammatory responses.

### 3.6 In Vivo NPY1R Intervention Modulates CREB Signaling Activation in Ovarian Follicles and Reverses Ovarian Inflammatory Phenotype

**FIGURE 6:**
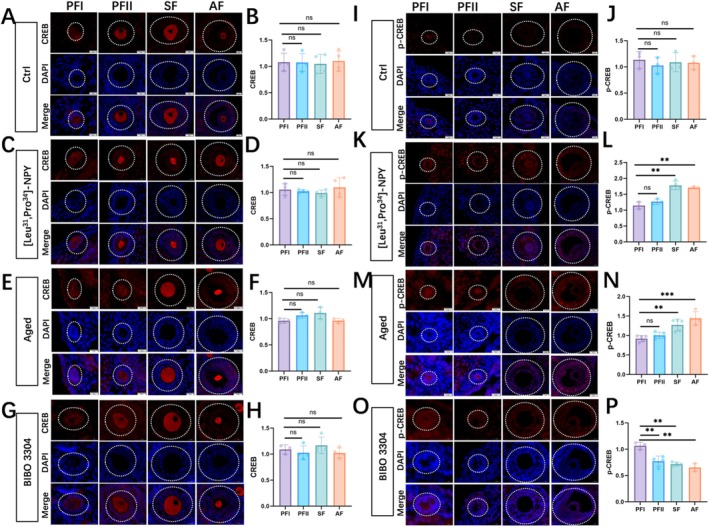
Effect of in vivo NPY1R intervention on CREB signaling activation in mouse ovarian follicles. (A–H) Expression and localization of CREB in ovarian follicles. CREB expression in ovarian sections of young control (Ctrl, A, B), young + agonist ([Leu^31^, Pro^34^]‐NPY, C, D), aged control (Aged, E, F), and aged + antagonist (BIBO 3304, G, H) mice was detected via immunofluorescence staining. Histograms show quantitative analysis of fluorescence intensity in follicles at each stage (PFI: Primordial follicle; PFII: Primary follicle; SF: Secondary follicle; AF: Antral follicle). (I–P) Expression and localization of p‐CREB in ovarian follicles. p‐CREB (phosphorylated CREB) expression in ovarian sections of mice from each group was detected via immunofluorescence staining (I–P). Histograms show quantitative analysis of p‐CREB fluorescence intensity in follicles at each stage. Image scale bars from left to right: 10, 20, 20, 50 μm. ns, no difference; **p < 0.01, ***p < 0.001. Data are presented as mean ± SD.

To verify the regulatory effect of NPY1R on the CREB signaling pathway in vivo, the expression and localization of CREB and its phosphorylated form (p‐CREB) in ovarian follicles of mice from different treatment groups were analyzed via immunofluorescence staining. In the ovaries of normal young mice (Ctrl), CREB protein was widely localized in granulosa cells and oocytes of follicles at all stages (primordial follicles PFI, primary follicles PFII, secondary follicles SF, antral follicles AF), with strong fluorescence signals in oocytes and no significant difference in expression intensity among follicle stages (*p* > 0.05) (Figure 6A,B). The localization pattern of CREB in the ovaries of aged mice (Aged) was similar to that of the young control group (Figure 6E,F; Figure S2). Neither treatment of young mice with NPY1R agonist [Leu^31^, Pro^34^]‐NPY (Figure 6C,D) nor treatment of aged mice with NPY1R antagonist BIBO 3304 (Figure 6G,H) significantly altered the expression level and localization pattern of CREB in follicles at all stages (*p* > 0.05).

However, the expression profile of p‐CREB (activated CREB) exhibited significant changes. In the ovaries of normal young mice, p‐CREB was predominantly expressed in granulosa cells of follicles at all stages, with weak or undetectable expression in oocytes, and no difference in expression level among follicle stages (*p* > 0.05) (Figure 6I,J). In contrast, the overall p‐CREB fluorescence signal was enhanced in the ovaries of aged mice, and its expression was significantly upregulated starting from the secondary follicle (SF) stage (*p* < 0.01) (Figure 6M,N; Figure S2B). NPY1R agonist treatment mimicked this aging‐related phenotype, significantly enhancing p‐CREB signals in SF and AF stage follicles of young mouse ovaries (*p* < 0.01) (Figure 6K,L). Conversely, NPY1R antagonist BIBO 3304 treatment effectively reversed the aberrant activation of p‐CREB in aged mouse ovaries, significantly inhibiting its expression in PFII, SF, and AF stages (*p* < 0.01) (Figure 6O,P). These results suggest that NPY1R‐mediated CREB signaling activation mainly occurs in follicular granulosa cells and is closely related to follicular development and aging status.

**FIGURE 7:**
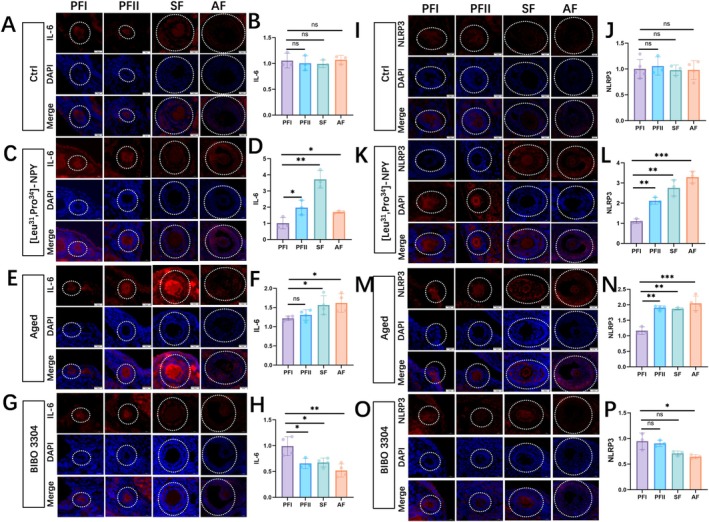
Effect of in vivo NPY1R intervention on inflammatory factor expression in mouse ovarian follicles. (A–H) Expression of IL‐6 in ovarian follicles. IL‐6 expression in ovarian sections of mice from each group was detected via immunofluorescence staining (A–H). Histograms show quantitative analysis of IL‐6 fluorescence intensity in follicles at each stage. (I–P) Expression of NLRP3 in ovarian follicles. NLRP3 expression in ovarian sections of mice from each group was detected via immunofluorescence staining (I–P). Histograms show quantitative analysis of NLRP3 fluorescence intensity in follicles at each stage. Image scale bars from left to right: 10 μm, 20 μm, 20 μm, 50 μm. Data are presented as mean ± SD. *p < 0.05, **p < 0.01, ***p < 0.001; ns, no statistically significant difference.

Based on the previous finding that NPY1R regulates the expression of inflammatory factors IL‐6 and NLRP3 in granulosa cells, we further investigated the effect of NPY1R intervention on the local ovarian inflammatory phenotype in vivo. In the ovaries of normal young mice, the expression levels of IL‐6 and NLRP3 were low, mainly distributed in granulosa cells with barely detectable signals in oocytes, and no significant differences among follicle stages (*p* > 0.05) (Figure 7A,B,I,J). NPY1R agonist treatment significantly induced the upregulation of IL‐6 and NLRP3 expression in young mouse ovaries (*p* < 0.05), and NLRP3 expression gradually increased with follicular development (Figure 7C,D,K,L). In the ovaries of aged mice, an obvious inflammatory phenotype was observed: the expression levels of IL‐6 and NLRP3 were significantly elevated with follicular development (*p* < 0.05), and clear positive signals were also present in oocytes (Figure 7E,F,M,N). Quantitative analysis showed that IL‐6 expression was significantly higher in aged mice than in young controls starting from the SF stage (*p* < 0.05) (Figure S2C), and NLRP3 expression was significantly upregulated in follicles at all stages (*p* < 0.05) (Figure S2C). Importantly, NPY1R antagonist BIBO 3304 treatment significantly reversed the upregulation trend of IL‐6 and NLRP3 during follicular development in aged mice (*p* < 0.05) (Figure 7G,H,O,P).

The above in vivo experimental results indicate that NPY1R may promote the expression of inflammatory factors IL‐6 and NLRP3 by upregulating p‐CREB levels in follicular granulosa cells, ultimately participating in the modulation of the aging‐related ovarian inflammatory microenvironment and follicular development process.

### 3.7 Aberrant NPY1R Activation Exacerbates Ovarian Tissue Inflammation and Fibrosis

**FIGURE 8:**
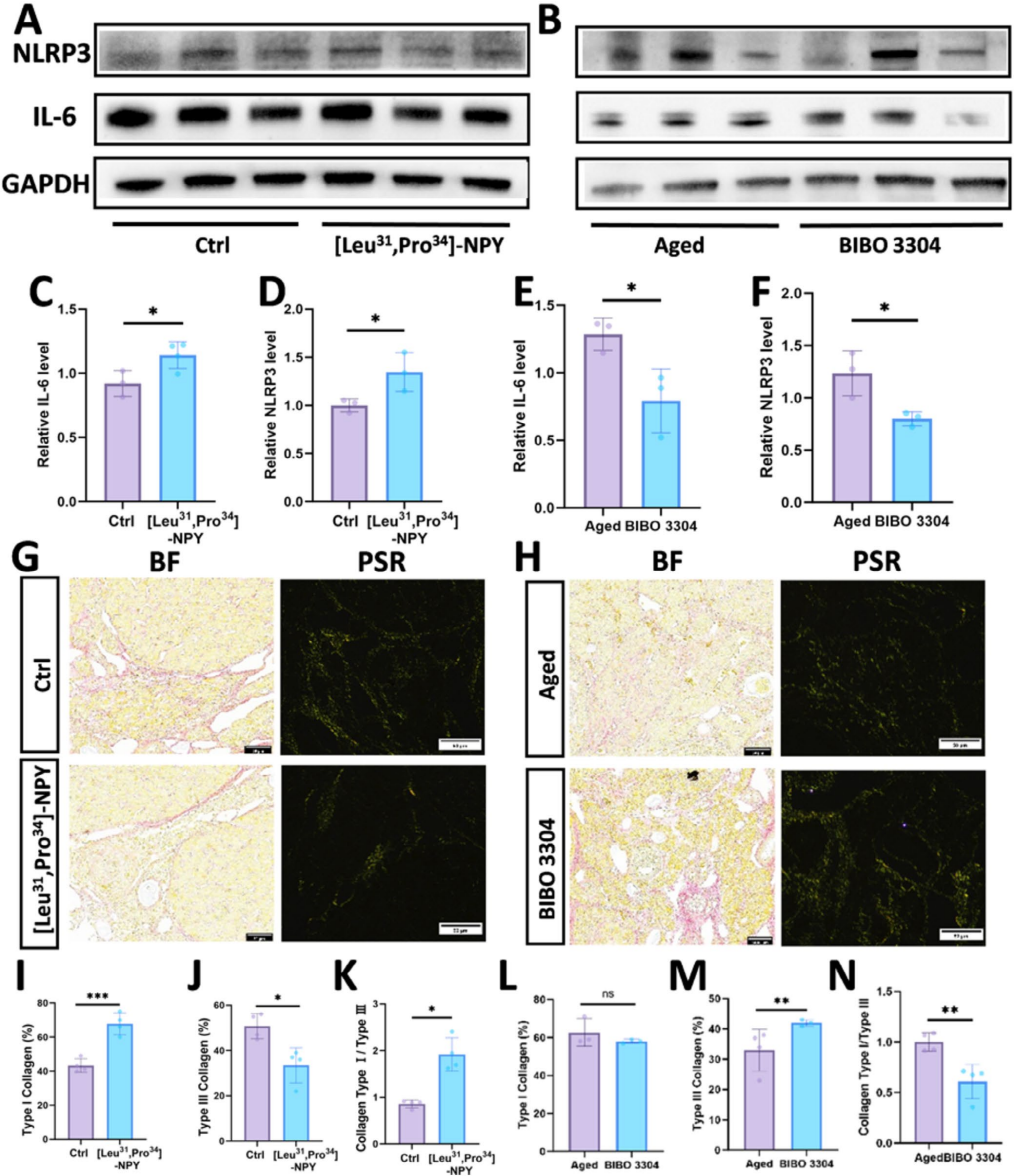
Effect of in vivo NPY1R pathway intervention on ovarian tissue inflammation and fibrosis in mice. (A–F) After feeding, ovarian tissues were collected from 3 mice in each of the four groups, total protein was extracted, and protein expression levels of IL‐6 and NLRP3 were detected via Western Blot (A, B: Western Blot band diagrams; C, E: Quantitative analysis of IL‐6 protein; D, F: Quantitative analysis of NLRP3 protein). (G–N) Picrosirius Red (PSR) staining combined with polarized light microscopy was used to evaluate the degree of ovarian tissue fibrosis in the Ctrl, Ctrl + [Leu^31^, Pro^34^]‐NPY, Aged, and Aged + BIBO 3304 groups. (G, H) Typical bright‐field and PSR staining images (under polarized light, type I collagen appears bright yellow/orange‐red, type III collagen appears bright green). (I–K) Quantitative analysis of PSR staining results in the Ctrl and Ctrl + [Leu^31^, Pro^34^]‐NPY groups: Proportion of type I collagen (I), proportion of type III collagen (J), type I/III collagen ratio (K). (L–N) Quantitative analysis of PSR staining results in the Aged and Aged + BIBO 3304 groups: Proportion of type I collagen (L), proportion of type III collagen (M), type I/III collagen ratio (N). Data are presented as Mean ± SD. *p < 0.05, **p < 0.01, ***p < 0.001; ns, no statistically significant difference.

To clarify the regulatory effect of NPY1R on ovarian tissue inflammation and fibrosis, ovarian tissues of mice from four groups were collected after treatment, and the protein expression levels of inflammation‐related genes were detected and fibrosis degree was evaluated. Western Blot results showed that the protein expression levels of IL‐6 and NLRP3 were significantly increased in ovarian tissues of mice in the NPY1R agonist‐treated group (Ctrl+[Leu^31^, Pro^34^]‐NPY) compared with the young control group (Ctrl) (*p* < 0.05) (Figure 8A,C,D). In contrast, the protein expression levels of IL‐6 and NLRP3 were significantly decreased in ovarian tissues of mice in the NPY1R antagonist‐treated group (Aged + BIBO 3304) compared with the aged control group (Aged) (*p* < 0.05) (Figure 8B,E,F), suggesting that aberrant NPY1R activation promotes ovarian tissue inflammation, while its antagonism effectively alleviates the ovarian inflammatory phenotype in aged mice.

Given that aberrant expression of inflammatory factors in ovarian granulosa cells may exert profound effects on ovarian function, Picrosirius Red (PSR) staining combined with polarized light microscopy was further used to systematically evaluate the regulatory effect of aberrant NPY1R activation on ovarian tissue fibrosis in mice. Under polarized light, type I collagen fibers show strong birefringence (bright yellow or orange‐red) with coarse and tightly arranged structures, while type III collagen fibers exhibit weak birefringence (bright green) with fine and loosely arranged structures, which can be clearly distinguished by color differences. In ovarian tissues of young control mice (Ctrl), collagen fibers were regularly distributed and moderately abundant, mainly localized around follicles in the ovarian cortex (forming delicate theca interstitium) and around blood vessels in the medulla, presenting sparse and slender red fiber bundles without collagen fiber wrapping around follicles or diffuse interstitial filling (Figure 8G). In contrast, the weak birefringence bright green signal corresponding to type III collagen was significantly enhanced in the ovaries of aged control mice (Aged), with fine and loose fibers widely distributed in the theca layer around follicles and ovarian stroma (Figure 8H). Quantitative analysis showed that compared with the Ctrl group, the proportion of type I collagen was significantly increased (*p* < 0.001), the proportion of type III collagen was significantly decreased (*p* < 0.01), and the type I/III collagen ratio was significantly elevated (*p* < 0.01) in ovarian tissues of aged mice (Figure S3), indicating aggravated ovarian fibrosis and imbalanced collagen fiber type distribution in aged mice.

To explore the regulatory role of NPY1R, young mice were treated with NPY1R agonist [Leu^31^, Pro^34^]‐NPY. The results showed that collagen fibers were significantly increased in ovarian tissues of the agonist‐treated group, presenting coarse and dense red cord‐like structures diffusely distributed and filling the cortical stroma, with thickened collagen fibers encapsulating follicles, exhibiting a fibrotic phenotype similar to that of aged mice (Figure 8G). Quantitative analysis showed that compared with the Ctrl group, the proportion of type I collagen was significantly increased (*p* < 0.001), the proportion of type III collagen was significantly decreased (*p* < 0.05), and the type I/III collagen ratio was significantly elevated (*p* < 0.05) in the [Leu31, Pro34]‐NPY group (Figure 8I–K), confirming that NPY1R agonist mimics the ovarian fibrotic phenotype of aged mice and exacerbates ovarian fibrosis. Furthermore, aged mice were treated with NPY1R antagonist BIBO 3304 to investigate its reversing effect on ovarian fibrosis. The results showed that ovarian fibrosis was significantly improved in the antagonist‐treated group, with uniformly distributed strong birefringent bright yellow/orange‐red type I collagen, stable type I/III collagen ratio, and no abnormal collagen fiber hyperplasia or loss, with clear contrast to background tissues (Figure 8H). Quantitative analysis showed that compared with the Aged group, the proportion of type I collagen showed no significant difference (*p* > 0.05), the proportion of type III collagen was significantly increased (*p* < 0.01), and the type I/III collagen ratio was significantly decreased (*p* < 0.01) in the BIBO 3304 group (Figure 8L–N), indicating that NPY1R antagonist effectively reverses ovarian fibrosis in aged mice.

In summary, these results demonstrate that aberrant NPY1R activation exacerbates ovarian tissue inflammation and fibrosis by regulating imbalanced collagen fiber type distribution, while its specific antagonist BIBO 3304 effectively reverses this phenotype, further corroborating the key regulatory role of NPY1R in the ovarian aging process.

## 4 Discussion

Ovarian aging, a progressive physiological process accompanying advancing female age, is characterized by depleted follicular reserve, declined oocyte quality, markedly elevated pro‐inflammatory factors, increased collagen deposition, enhanced oxidative stress, and mitochondrial dysfunction [10]. Sustained abnormal ovarian inflammation establishes a chronic pro‐inflammatory microenvironment, which impairs oocytes' developmental and maturation competence [10]; moreover, the inflammatory response can further induce ovarian oxidative stress and tissue fibrosis [30]. Previous studies have demonstrated that in patients with polycystic ovary syndrome (PCOS), increased levels of inflammatory factors (such as CRP, IL‐6, TNF‐α, IL‐1β) and excessive reactive oxygen species (ROS) accumulation in follicular fluid promote the deposition of ovarian lipid peroxidation products and compromise the ovarian antioxidant defense system. Consequently, the quantity of high‐quality oocytes is reduced, and infertility may occur; these pathological changes are particularly severe in obese individuals with concomitant PCOS [31]. In addition, the levels of pro‐inflammatory cytokines in follicular fluid of patients with endometriosis are usually elevated, which can induce follicular oxidative stress and fibrosis, thereby interfering with the developmental process of oocytes [32]. During the aging process, senescence‐associated secretory phenotype (SASP) factors secreted by senescent cells induce inflammatory responses and oxidative stress in surrounding cells. The levels of pro‐inflammatory factors (such as IL‐6, IL‐1β, TNF‐α) in the serum of advanced‐age women are significantly upregulated to varying degrees, which may damage follicular development or reduce oocyte quality by establishing a pro‐inflammatory and stressful microenvironment [9]. Therefore, regulating the process of ovarian inflammation and fibrosis is expected to become a novel therapeutic strategy to improve the follicular microenvironment and alleviate ovarian dysfunction.

To explore the molecular mechanism of advanced‐age ovarian aging, this study first compared the gene expression profiles of granulosa cells derived from patients with normal ovarian function and aged patients. It was found that a large number of differentially expressed genes existed in granulosa cells of the advanced‐age group, and these genes were mainly enriched in the cAMP signaling pathway, suggesting that the abnormal cAMP pathway may participate in the process of ovarian aging. Among them, *NPY1R*, a key candidate gene in the cAMP pathway, was significantly upregulated in granulosa cells of elderly patients, and this finding was further confirmed by quantitative real‐time polymerase chain reaction (qPCR) results, indicating that *NPY1R* may be involved in the regulation of aging‐related ovarian dysfunction. In aged patients, the abnormally high expression of *NPY1R* may further exacerbate ovarian dysfunction by regulating cellular metabolism and proliferation. At present, the specific mechanism of *NPY1R* in ovarian granulosa cells has not been fully elucidated, but existing studies have confirmed its crucial role in ovarian function regulation: studies in porcine ovarian granulosa cells have shown that neuropeptide Y (NPY) can inhibit cell proliferation, promote apoptosis, and lead to intracellular accumulation of p53 protein [33]; moreover, an increasing number of experiments have verified the presence of NPY receptors in human ovarian cells [34], and these receptors are involved in the regulation of female reproductive function [35]. The NPY family comprises five receptors, among which Y1 and Y4 exert more significant regulatory effects on the female reproductive system than Y2, Y5, and Y6, with the Y1 receptor negatively regulating ovarian reserve [36] and the Y4 receptor positively regulating it [37]; notably, the affinity of NPY for the Y1 receptor is 1000 times higher than that for the Y4 receptor [18, 38]. Another study found that quercetin injection in mice reduced NPY expression levels, while significantly increasing the number of follicles and fertility in mice [39], further corroborating the important role of NPY and its receptors in ovarian function regulation.

To clarify the regulatory effect of *NPY1R* on ovarian function, this study administered dietary intervention with *NPY1R* agonist ([Leu^31^, Pro^34^]‐NPY) or antagonist (BIBO 3304) to normal young mice and aged mice respectively, and systematically evaluated their impacts on ovarian function and oocyte quality in mice. The results showed that mice in the [Leu^31^, Pro^34^]‐NPY treatment group exhibited significant ovarian dysfunction, manifested as estrous cycle disorders and abnormal follicular development; in contrast, ovarian function in mice of the BIBO 3304 treatment group was significantly reversed, with the estrous cycle returning from disorder to normal and the number of ovarian follicles increasing markedly. These findings suggest that activation of *NPY1R* may impair follicular development and reserve by interfering with reproductive endocrine function, ultimately leading to diminished ovarian reserve. Previous studies have shown that NPY, as a vital neuroendocrine regulatory factor [22], plays a key role in both the nervous and reproductive systems, while the reproductive endocrine system is mainly regulated by the hypothalamic–pituitary‐gonadal (HPG) axis. NPY can inhibit the excitability of GnRH neurons through Y1 receptor, thereby exerting a negative regulatory effect on the HPG axis [18], which is consistent with the results of *NPY1R* agonist‐induced ovarian dysfunction in this study. The endogenous ligand NPY exhibits approximately 1000‐fold higher affinity for the NPY1R relative to the NPY Y4 receptor [18, 38]. However, the agonist [Leu^31^, Pro^34^]‐NPY and antagonist BIBO 3304 used in this study are highly selective pharmacological tools for NPY1R, with negligible cross‐reactivity toward other NPY receptor subtypes including Y4. Therefore, the 1000‐fold affinity difference between NPY1R and Y4 does not affect the receptor selectivity or mechanistic interpretation of our findings.

Oocyte quality is an important indicator for ovarian function, and mitochondria, as the core of cellular energy metabolism, are critical for oocyte maturation and fertilization capacity, whose structural and functional integrity is essential [40, 41]. This study found that *NPY1R* agonist diet led to a significant increase in ROS levels and impaired mitochondrial function in mouse oocytes, manifested as abnormal mitochondrial morphology (swelling, cristae fragmentation) under transmission electron microscopy (TEM) and a significantly elevated proportion of abnormal mitochondria; conversely, *NPY1R* antagonist diet reversed the abnormal ROS levels and mitochondrial dysfunction in oocytes, with a significantly increased proportion of normal mitochondria, further confirming the negative impact of *NPY1R* activation on oocyte quality.

Recent studies have demonstrated that ferroptosis is closely associated with ovarian follicular development. Elevated oxidative stress levels in the ovary during aging can induce ROS accumulation and decreased mitochondrial membrane potential, thereby triggering follicular atresia and oocyte dysfunction, and ultimately inducing ferroptosis [42]. Electron microscopic analysis in the present study showed that oocyte mitochondria in the [Leu^31^, Pro^34^]‐NPY‐treated group and aged (Aged) group exhibited abnormal morphology, with a significantly higher proportion of abnormal mitochondria; in contrast, mitochondrial morphology in the normal control (Ctrl) group and BIBO 3304 treatment group tended to be normal, with a significantly reduced proportion of abnormal mitochondria, verifying the core role of mitochondrial dysfunction in the process of ferroptosis. Meanwhile, the intracellular Fe^2+^ level in oocytes of mice in the [Leu^31^, Pro^34^]‐NPY treatment group was significantly increased, suggesting that agonist diet may accelerate oxidative damage and mitochondrial dysfunction by promoting iron ion accumulation; whereas the intracellular Fe^2+^ level in oocytes of mice in the BIBO 3304 treatment group was significantly decreased, indicating that the antagonist can reduce iron accumulation in oocytes by downregulating *NPY1R* expression, thereby alleviating oxidative damage and reversing mitochondrial dysfunction. These results indicate that aberrant *NPY1R* expression may induce mitochondrial dysfunction by regulating oxidative stress and iron metabolism disorders, thereby impairing oocyte quality and reducing ovarian function, which also provides a new theoretical basis for the pathogenesis of aging‐related infertility.

Aging is often accompanied by abnormally high expression of inflammatory factors. Based on this, this study further verified the expression characteristics of inflammatory factors in ovarian granulosa cells and found that the expression levels of *IL‐6* and *NLRP3* in granulosa cells derived from elderly patients were significantly higher than those in the normal control group. After *NPY1R* pharmacological intervention, the expression of *IL‐6* and *NLRP3* in granulosa cells of the [Leu^31^, Pro^34^]‐NPY treatment group was significantly increased, while that in the BIBO 3304 treatment group was significantly decreased, indicating that NPY1R can regulate the expression of inflammatory factors during aging. This result is consistent with the in vivo mouse experimental findings: immunofluorescence staining of mouse ovarian sections showed that the expression of *IL‐6* in the middle and late stages of follicular development and *NLRP3* throughout the entire follicular development process was significantly increased in the Aged group. During follicular maturation, *IL‐6*, as a local regulatory factor, can promote the proliferation and differentiation of granulosa cells and oocyte maturation, but excessive elevation of *IL‐6* activates inflammatory pathways, leading to follicular developmental arrest and reduced oocyte quality. In PCOS models, elevated *IL‐6* exacerbates androgen disorders and further hinders follicular maturation [43], while reduced *IL‐6* levels in follicular fluid during in vitro culture can significantly improve pregnancy rates [44]. Excessive activation of NLRP3 releases a large number of pro‐inflammatory factors (such as IL‐1β and IL‐18), causing ovarian tissue damage and follicular developmental arrest [45, 46]; sustained activation further results in massive loss of oocytes and granulosa cells, severely impairing reproductive function [46, 47].

This study further explored the molecular mechanism by which *NPY1R* regulates ovarian inflammation and function and found that the expression pattern of genes related to the cAMP‐CREB signaling pathway was significantly altered in granulosa cells of aging patients, suggesting that *NPY1R* may participate in the regulation of aging‐related ovarian dysfunction by modulating the cAMP‐CREB signaling pathway [21, 48]. In granulosa cells of aging patients, upregulated *NPY1R* expression may be closely associated with aging‐induced increased inflammatory factors and ovarian dysfunction, while the *NPY1R* antagonist BIBO 3304 can inhibit the release of inflammatory factors by downregulating *NPY1R* expression, helping to restore the normal function of granulosa cells and alleviate aging‐induced ovarian dysfunction. Both western blot and qPCR results confirmed that the expression levels of *NPY1R*, p‐CREB/CREB, *IL‐6*, and *NLRP3* were significantly increased in granulosa cells of aged patients, further corroborating the involvement of *NPY1R* in the regulation of advanced‐age related ovarian dysfunction.

Inflammatory response is a crucial driver of ovarian fibrosis [10]. Excessive secretion of pro‐inflammatory factors activates fibroblasts and immune cells, promotes aberrant deposition of collagen and other extracellular matrix (ECM) components, and ultimately leads to ovarian tissue sclerosis and ovarian dysfunction [49]. This mechanism is consistent with the experimental results of mouse ovaries in this study: the proportion of type I collagen and the type I/III collagen ratio were significantly increased in the ovaries of mice in the [Leu^31^, Pro^34^]‐NPY treatment group, indicating that agonist diet can induce high expression of inflammatory factors by promoting *NPY1R* expression, thereby triggering ovarian fibrosis, disrupting the ovarian microenvironment, and ultimately impeding follicular development; in contrast, the ovarian fibrotic lesions in mice of the BIBO 3304 treatment group were significantly reversed, indicating that antagonist diet can improve the ovarian microenvironment by downregulating *NPY1R* expression and reducing inflammatory factor levels, thus facilitating normal follicular development and maturation. Notably, there is a vicious cycle between inflammation and ovarian fibrosis: inflammation initiates the fibrotic process via pro‐inflammatory factors, while fibrosis exacerbates local inflammatory responses and further disrupts normal ovarian physiological functions [50].

The theoretical significance of this study lies in systematically elucidating the regulatory role and molecular mechanism of the *NPY1R*/CREB signaling axis in the inflammatory response of aged granulosa cells, refining the molecular regulatory network underlying ovarian aging, and providing new theoretical support for research in the field of reproductive aging. By establishing the association between *NPY1R* and the inflammatory response of aged granulosa cells, this study clarifies that abnormally high *NPY1R* expression can exacerbate ovarian dysfunction, while *NPY1R* antagonists can reverse the inflammatory phenotype of granulosa cells and ameliorate age‐induced ovarian dysfunction by downregulating its expression. This study is expected to provide a novel intervention strategy for improving the reproductive outcomes of advanced‐age women: by identifying the *NPY1R*‐CREB signaling axis as a potential intervention target, specific inhibitors or activators can be developed and applied in assisted reproductive technology to improve granulosa cell function and oocyte quality, and enhance treatment success rates; in addition, molecular markers such as *NPY1R* identified in this study can be used to evaluate ovarian reserve function and reproductive prognosis in elderly women, providing a reference for the establishment of personalized clinical therapeutic strategies. BIBO 3304 is a highly selective and well‐characterized NPY1R antagonist with favorable safety and tolerability in preclinical animal studies, supporting its promising translational potential for future development in ovarian protection and age‐related reproductive disorders.

In conclusion, the inflammatory response of aged granulosa cells is a critical mechanism underlying ovarian function decline, and the *NPY1R*‐CREB signaling axis exerts a pivotal regulatory role in this process. Although *NPY1R* antagonism has a significant effect on improving ovarian function in aged mice, abnormally elevated *NPY1R* activity may attenuate this interventional effect. Future studies can further explore potential targeted therapeutic strategies for *NPY1R* antagonism to more effectively ameliorate *NPY1R‐overexpression‐related* ovarian dysfunction. Meanwhile, the clinical sample size can be expanded to validate the clinical value of *NPY1R* as a molecular marker and therapeutic target for ovarian aging.

## Author Contributions

J.F., C.W., and S.G. are responsible for most of the experimental research, data analysis and draft writing. Y.K., Yiping Zhang, and Yang Zhang are responsible for part of the experimental research and data analysis, H.J., L.Q., Y.L., Z.W., F.Z., S.W., and X.W. contributed to sample collection and writing suggestion. G. Yang is responsible for experimental design, research supervision and data analysis. G. Yao is responsible for conceptualization, methodology, supervision, funding acquisition, project administration, writing review and editing, final approval, communication with journal. All authors reviewed the manuscript.

## Funding

This work was supported by the following grants: the Natural Science Foundation of Henan Province (262300421646), the Key Scientific Research Projects of Henan Higher Education Institutions (26A320024), the Provincial and Ministerial Co‐construction Project of Henan Medical Science and Technology Program (SBGJ202502063), the Open Research Fund of the National Health Commission Key Laboratory of Birth Defects Prevention (NHCKLBDP202407), and the Henan Provincial Science and Technology R&D Program Joint Fund Project (LHGJ20250284).

## Conflicts of Interest

The authors declare no conflicts of interest.

## Supporting information


**Figure S1:** Validation of NPY1R overexpression and knockdown efficiency. (A, B) Validation of NPY1R overexpression efficiency. KGN cells were transfected with adenovirus‐mediated control (Ctrl) or NPY1R overexpression (Oe‐NPY1R) vectors for 48 h; NPY1R protein expression was detected via Western Blot (A) and gray value quantitative analysis was performed (B). GAPDH was used as the internal reference protein. (C–E) Validation of NPY1R knockdown efficiency. Human primary granulosa cells were transfected with control siRNA (si‐Ctrl) or NPY1R‐specific siRNA (si‐NPY1R); mRNA expression level of NPY1R (C) and protein expression level of NPY1R (D) were detected via qPCR and Western Blot respectively, followed by gray value quantitative analysis (E). Data are presented as mean ± SD. ****p < 0.0001; different letters at the top of histograms indicate significant differences.

**Figure S2:** Comparison of CREB, p‐CREB, IL‐6, and NLRP3 expression in ovarian follicles of young and aged mice. (A) Comparison of CREB expression. Quantitative statistics of CREB fluorescence intensity in follicles at each stage of ovaries from young control and aged control mice. (B) Comparison of p‐CREB expression. Quantitative statistics of p‐CREB fluorescence intensity in follicles at each stage of ovaries from young control and aged control mice. (C) Comparison of IL‐6 expression. Quantitative statistics of IL‐6 fluorescence intensity in follicles at each stage of ovaries from young control and aged control mice. (D) Comparison of NLRP3 expression. Quantitative statistics of NLRP3 fluorescence intensity in follicles at each stage of ovaries from young control and aged control mice. PFI: primordial follicle; PFII: primary follicle; SF: secondary follicle; AF: antral follicle. Data are presented as mean ± SD. *p < 0.05, **p < 0.01, ***p < 0.001; ns, no statistically significant difference.

**Figure S3:** Comparison of ovarian tissue fibrosis between young control and aged mice. (A–C) Quantitative analysis of the proportion of type I collagen (A), proportion of type III collagen (B), and type I/III collagen ratio (C) in ovarian tissues of 8‐week‐old (Ctrl) and 12‐month‐old (Aged) mice via PSR staining. Data are presented as mean ± SD. **p < 0.01, ***p < 0.001.

**Table S1:** The primers used in this research.

## Data Availability

All data generated or analyzed during this study are included in this published article and its Supporting Information files.
