# Supplementary figures and images for: Mechanism and Intervention of the NPY1R/CREB Signaling Axis in Regulating Inflammatory Response in Aged Ovarian Granulosa Cells and Ovarian Senescence
Source: FASEB J. 2026 Jun 9;40(12):e72026. doi: 10.1096/fj.202601614R (PMC13249023; doi:10.1096/fj.202601614R)

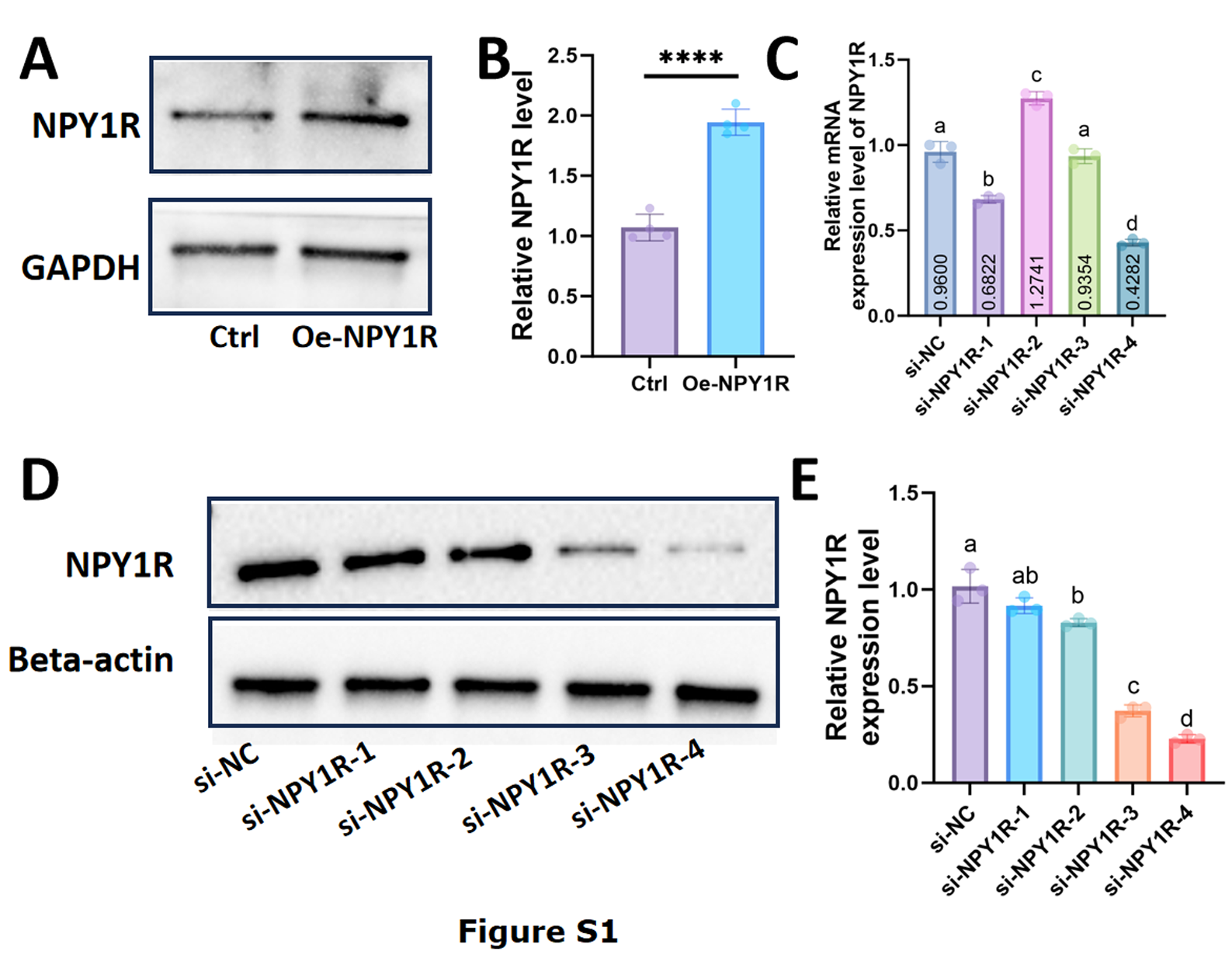

Supplement: Supplementary file 1 — Figure S1: Validation of NPY1R overexpression and knockdown efficiency. (A, B) Validation of NPY1R overexpression efficiency. KGN cells were transfected with adenovirus‐mediated control (Ctrl) or NPY1R overexpression (Oe‐NPY1R) vectors for 48 h; NPY1R protein expression was detected via Western Blot (A) and gray value quantitative analysis was performed (B). GAPDH was used as the internal reference protein. (C–E) Validation of NPY1R knockdown efficiency. Human primary granulosa cells were transfected with control siRNA (si‐Ctrl) or NPY1R‐specific siRNA (si‐NPY1R); mRNA expression level of NPY1R (C) and protein expression level of NPY1R (D) were detected via qPCR and Western Blot respectively, followed by gray value quantitative analysis (E). Data are presented as mean ± SD. ****p < 0.0001; different letters at the top of histograms indicate significant differences. [file FSB2-40-e72026-s001.tif]

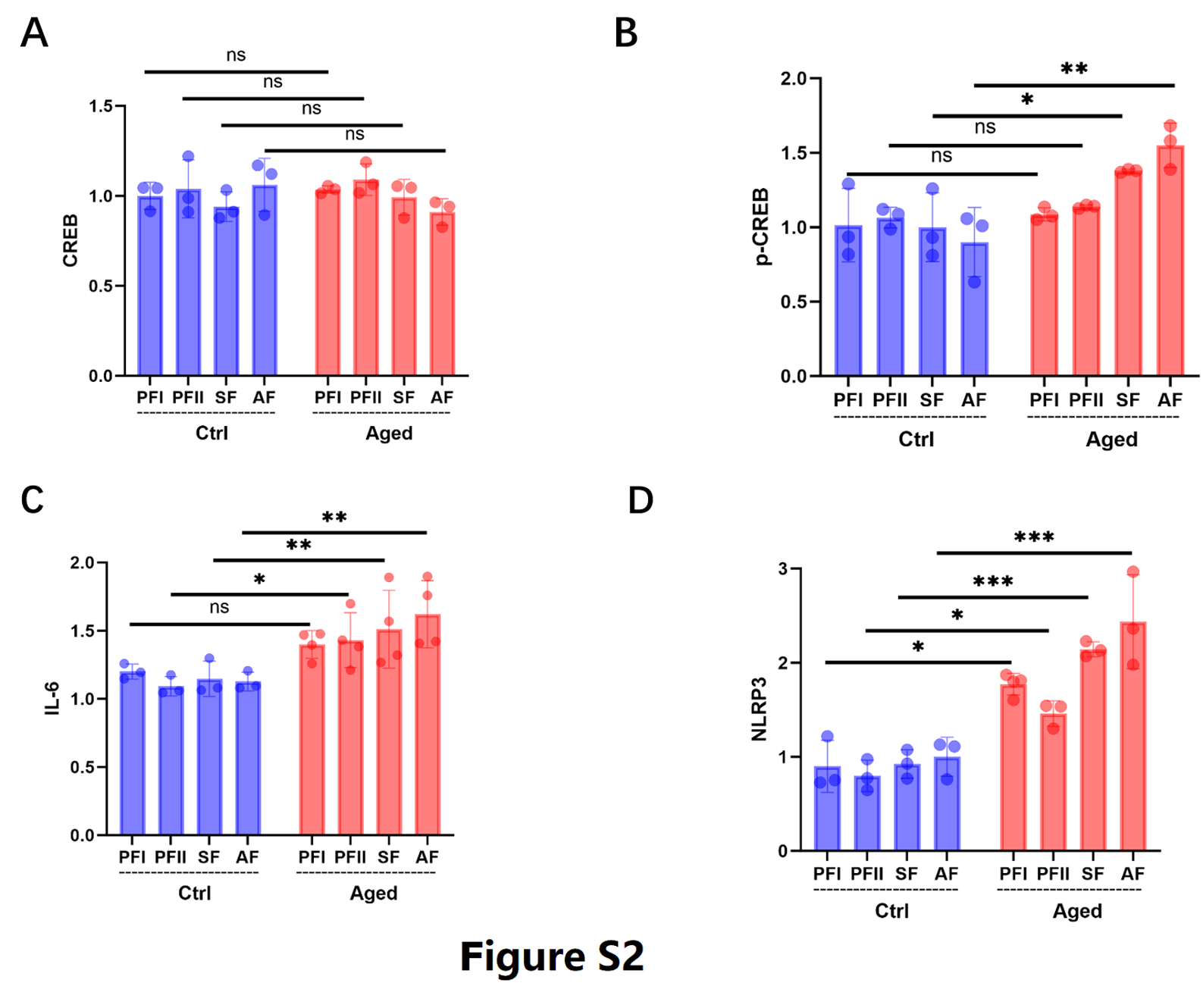

Supplement: Supplementary file 2 — Figure S2: Comparison of CREB, p‐CREB, IL‐6, and NLRP3 expression in ovarian follicles of young and aged mice. (A) Comparison of CREB expression. Quantitative statistics of CREB fluorescence intensity in follicles at each stage of ovaries from young control and aged control mice. (B) Comparison of p‐CREB expression. Quantitative statistics of p‐CREB fluorescence intensity in follicles at each stage of ovaries from young control and aged control mice. (C) Comparison of IL‐6 expression. Quantitative statistics of IL‐6 fluorescence intensity in follicles at each stage of ovaries from young control and aged control mice. (D) Comparison of NLRP3 expression. Quantitative statistics of NLRP3 fluorescence intensity in follicles at each stage of ovaries from young control and aged control mice. PFI: primordial follicle; PFII: primary follicle; SF: secondary follicle; AF: antral follicle. Data are presented as mean ± SD. *p < 0.05, **p < 0.01, ***p < 0.001; ns, no statistically significant difference. [file FSB2-40-e72026-s004.tif]

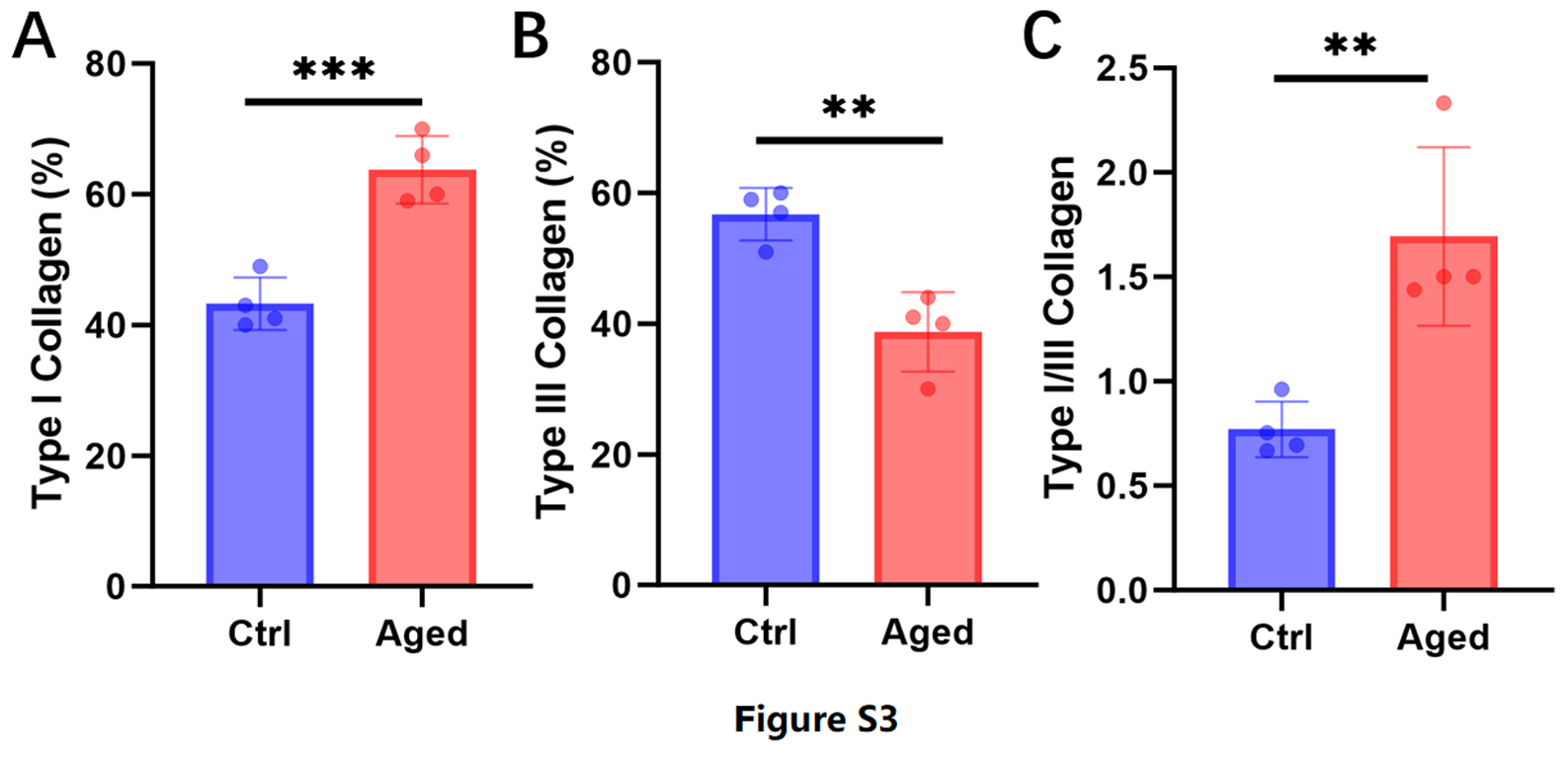

Supplement: Supplementary file 3 — Figure S3: Comparison of ovarian tissue fibrosis between young control and aged mice. (A–C) Quantitative analysis of the proportion of type I collagen (A), proportion of type III collagen (B), and type I/III collagen ratio (C) in ovarian tissues of 8‐week‐old (Ctrl) and 12‐month‐old (Aged) mice via PSR staining. Data are presented as mean ± SD. **p < 0.01, ***p < 0.001. [file FSB2-40-e72026-s003.tif]
