# Supplementary material for: Mechanism and Intervention of the NPY1R/CREB Signaling Axis in Regulating Inflammatory Response in Aged Ovarian Granulosa Cells and Ovarian Senescence
Source: FASEB J. 2026 Jun 9;40(12):e72026. doi: 10.1096/fj.202601614R (PMC13249023; doi:10.1096/fj.202601614R)
Supplement: Supplementary file 4 — Table S1: The primers used in this research. [file FSB2-40-e72026-s002.docx]

**Supplementary Table S1. The primers used in this research**

| **Gene name** |  | **Primer sequence** | **Product length** | **GenBank accession number** |
| --- | --- | --- | --- | --- |
| NPY1R(h) | F | TGCGGCGTTCAAGGACAAGTATG | 84 | NM_000909.6 |
|  | R | ACCAGGAGGAGAGTCGTGTAAGAC |  |  |
| EDN1(h) | F | TGCTCCTGCTCGTCCCTGATG | 99 | [NM_001168319.2](https://www.ncbi.nlm.nih.gov/nuccore/NM_001168319.2) |
|  | R | AAGTCCATACGGAACAACGTGCTC |  |  |
| CAMP(h) | F | ATGCTAACCTCTACCGCCTCCTG | 136 | [NM_004345.5](https://www.ncbi.nlm.nih.gov/nuccore/NM_004345.5) |
|  | R | CAATCCTCTGGTGACTGCTGTGTC |  |  |
| NAMPT(h) | F | TTCTGGAAACCCTCTTGACACTGTG | 98 | [NM_005746.3](https://www.ncbi.nlm.nih.gov/nuccore/NM_005746.3) |
|  | R | GTGGCAGCAACTTGTAACCCTTTG |  |  |
| AMH(h) | F | CCCGAGACACCCGCTACCTG | 146 | [NM_000479.5](https://www.ncbi.nlm.nih.gov/nuccore/NM_000479.5) |
|  | R | GTCGTCGCCGAACAGCAGTG |  |  |
| CXCL8(h) | F | CTCTCTTGGCAGCCTTCCTGATTTC | 116 | [NM_000584.4](https://www.ncbi.nlm.nih.gov/nuccore/NM_000584.4) |
|  | R | GGGGTGGAAAGGTTTGGAGTATGT |  |  |
| GBP5(h) | F | CCTCTATCGCACTGGCAAATCCTAC | 118 | [NM_001134486.4](https://www.ncbi.nlm.nih.gov/nuccore/NM_001134486.4) |
|  | R | AGGCACACACCATATCCAAATTCCC |  |  |
| PDE10A(h) | F | TGGAAACAGGAAGCAGTTGGAAGAG | 112 | [NM_001130690.3](https://www.ncbi.nlm.nih.gov/nuccore/NM_001130690.3) |
|  | R | ACAAAGGTCACAGGCAGTCATCATC |  |  |
| NPY2R(h) | F | TGGATGAACAGCAACTACAGAAAGG | 86 | [NM_000910.4](https://www.ncbi.nlm.nih.gov/nuccore/NM_000910.4) |
|  | R | ACGGACACCTCAGAGTGAATGG |  |  |
| NPY4R(h) | F | TTCATCGTCACTTCCTACAGCATTG | 120 | [NM_001278794.2](https://www.ncbi.nlm.nih.gov/nuccore/NM_001278794.2) |
|  | R | GTTGGCGATAAGCAGGTTGGTC |  |  |
| NPY5R(h) | F | ACGGTAAACTTCCTCATAGGCAATC | 104 | [NM_001317091.2](https://www.ncbi.nlm.nih.gov/nuccore/NM_001317091.2) |
|  | R | AACATCCACTGATCCAGCAAGAC |  |  |
| IL-6(h) | F | AGAGTAGTGAGGAACAAGCCAGAG | 119 | [NM_000600.5](https://www.ncbi.nlm.nih.gov/nuccore/NM_000600.5) |
|  | R | GGCATTTGTGGTTGGGTCAGG |  |  |
| NLRP3(h) | F | TGAGCACCAGCCAGAGTCTAAC | 110 | [NM_001079821.3](https://www.ncbi.nlm.nih.gov/nuccore/NM_001079821.3) |
|  | R | CCGAATGTTACAGCCAGGATGC |  |  |
| NPY1R(m) | F | CCACCAGATCATTGCCACCT | 170 | NM_001358955 |
|  | R | CATCGTCTCGAGACCGGAAG |  |  |
| IL-6(m) | F | GCCTTCTTGGGACTGATGCT | 124 | [NM_001314054.1](https://www.ncbi.nlm.nih.gov/nuccore/NM_001314054.1) |
|  | R | AGCCTCCGACTTGTGAAGTG |  |  |
| NLRP3(m) | F | AAACCCACCAGTGTGCAAGA | 86 | [NM_001359638.1](https://www.ncbi.nlm.nih.gov/nuccore/NM_001359638.1) |
|  | R | CAAAGGCCCCTTGTAGCTCA |  |  |
